# Tracing whale myoglobin evolution by resurrecting ancient proteins

**DOI:** 10.1038/s41598-018-34984-6

**Published:** 2018-11-15

**Authors:** Yasuhiro Isogai, Hiroshi Imamura, Setsu Nakae, Tomonari Sumi, Ken-ichi Takahashi, Taro Nakagawa, Antonio Tsuneshige, Tsuyoshi Shirai

**Affiliations:** 1 Department of Pharmaceutical Engineering, Toyama Prefectural University, Imizu, Toyama, 939-0398 Japan; 2 Department of Applied Chemistry, College of Life Sciences, Ritsumeikan University, 1-1-1 Nojihigashi, Kusatsu, Shiga 525-8577 Japan; 3 Department of Computer Bioscience, Nagahama Institute of Bio-Science and Technology, 1266 Tamura-Cho, Nagahama, Shiga 526-0829 Japan; 4 Research Institute for Interdisciplinary Science, Okayama University, 3-1-1 Tsushima-Naka, Kita-ku, Okayama 700-8530 Japan; 5 Department of Frontier Bioscience and Research Center for Micro-Nano Technology, Hosei University, Koganei, Tokyo Japan

## Abstract

Extant cetaceans, such as sperm whale, acquired the great ability to dive into the ocean depths during the evolution from their terrestrial ancestor that lived about 50 million years ago. Myoglobin (Mb) is highly concentrated in the myocytes of diving animals, in comparison with those of land animals, and is thought to play a crucial role in their adaptation as the molecular aqualung. Here, we resurrected ancestral whale Mbs, which are from the common ancestor between toothed and baleen whales (*Basilosaurus*), and from a further common quadrupedal ancestor between whale and hippopotamus (*Pakicetus*). The experimental and theoretical analyses demonstrated that whale Mb adopted two distinguished strategies to increase the protein concentration *in vivo* along the evolutionary history of deep sea adaptation; gaining precipitant tolerance in the early phase of the evolution, and increase of folding stability in the late phase.

## Introduction

The analysis of ancient proteins, experimentally reproduced by means of bioinformatics and genetic engineering, is a powerful tool for elucidating the biological molecular evolution and the relationships in protein sequence-structure-function. For example, the reconstruction of ancestral alcohol dehydrogenases from yeast revealed the connection between the chemical behavior of enzymes and the global ecosystem changes^1^. Functional analyses of ancient mammalian uricases demonstrated the evolutionary history of the enzyme and provided new therapeutics for human diseases^2^. The recreation of ancient fluorescent proteins revealed their photochemistry, which was applied to expand the variations of useful biological probes^3^. The determination of the ancestral structures of fish galectin revealed the atomic details of the functional differentiation process of the proteins^4^. The analysis of Precambrian β-lactamase demonstrated the molecular mechanism of novel active site formation^5^. The information obtained from these studies can be utilized in protein engineering and biomedical sciences. In the present study, we investigated the molecular evolution of whale Mb by experimentally resurrecting ancient proteins. The analyses of their chemical properties and structures demonstrated how Mb molecules evolved to adapt ancient whales to deep-sea environments.

Extant whales, such as sperm whales, acquired the great ability to dive into the ocean depths during the evolution from their terrestrial ancestor, which has been dated back to about 50 million years ago^6,7^. Their adaptation to the deep sea is thought to involve various physiological changes at the anatomical, cellular, and molecular levels^8–11^. Hemoglobin (Hb) and myoglobin (Mb) are the key molecules in animal aerobic exercise, as they are responsible for molecular oxygen (O_2_) transport in the bloodstream and its storage in the skeletal muscle, respectively. Thus, animal globins have been extensively studied and demonstrated to have evolved to adapt animals to their respective niches^12–18^. In the muscle tissues of deep diving animals, Mb is highly concentrated with its physiological function preserved, whereas the content is significantly lower in land animals, as shown in Table S1 and Fig. S1^8,11,19–22^. Thus, the diving capacity of mammals is thought to correlate with the Mb concentration in their myocytes. Recent studies suggested that the diving mammals have Mbs with more positive net surface charges (*Z*_Mb_) than those of terrestrial mammals, and predicted that ancient Mbs had less positive *Z*_Mb_ values than the offspring diving animals^11,21–24^. These positive charges have been expected to cause electrostatic repulsion among the Mb molecules, to prevent their aggregation and maintain the high protein concentration^21,25,26^.

**Figure 1:**
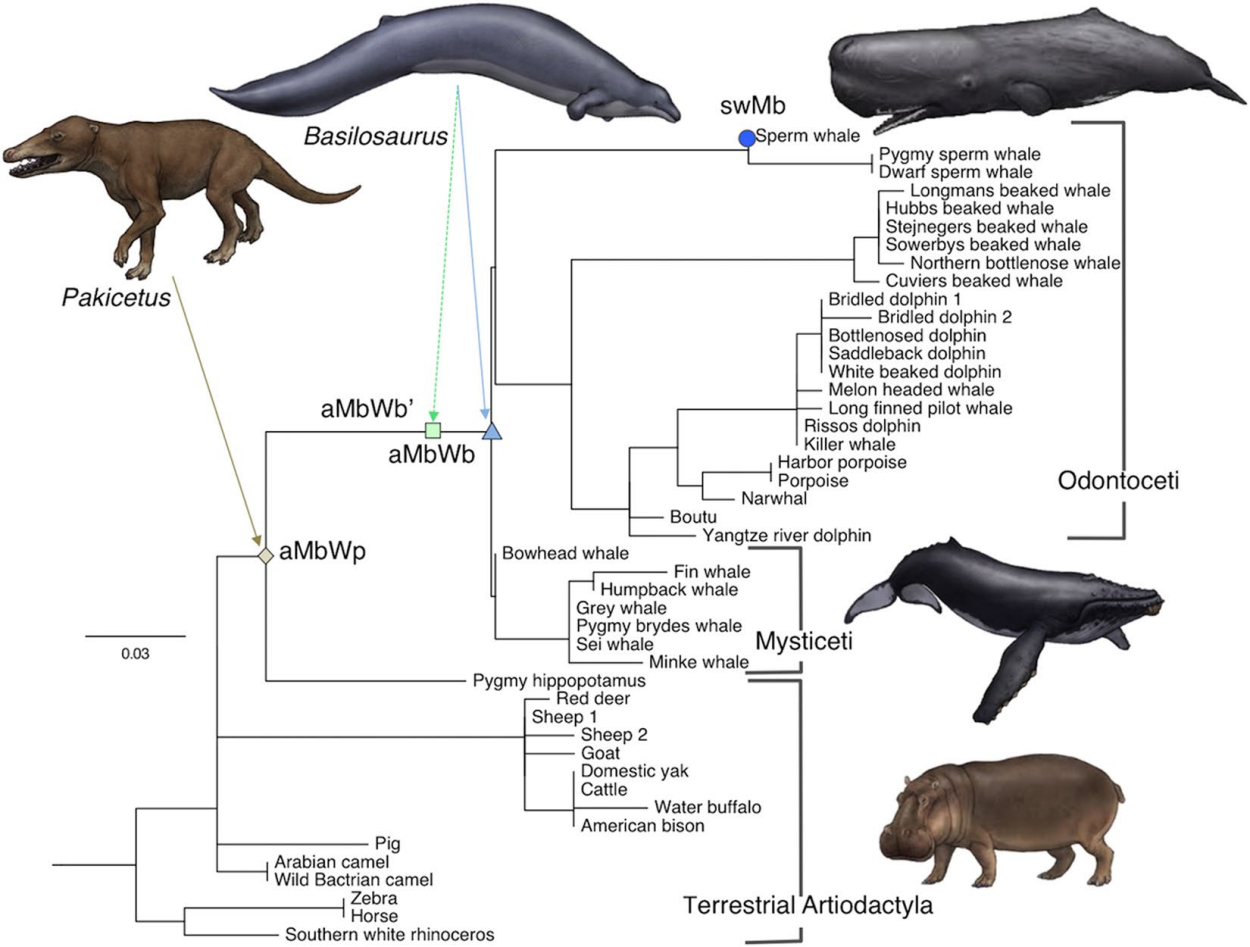
Molecular phylogenic tree of Mbs from whales, land animal relatives, and inferred ancestors. The presented tree is a part of the entire tree consisted of Hbs, Mbs, and other globins (Fig. S2a). Green, light blue, blue, and dark blue circles indicate the positions of aMbWp (land ancestor), aMbWb’ (polyphyly whale ancestor), aMbWb (monophyly whale ancestor), and extant swMb. The illustrations of animals are not covered by the CC BY license. Credit to Satoshi Kawasaki. All rights reserved, used with permission.

**Figure 2:**
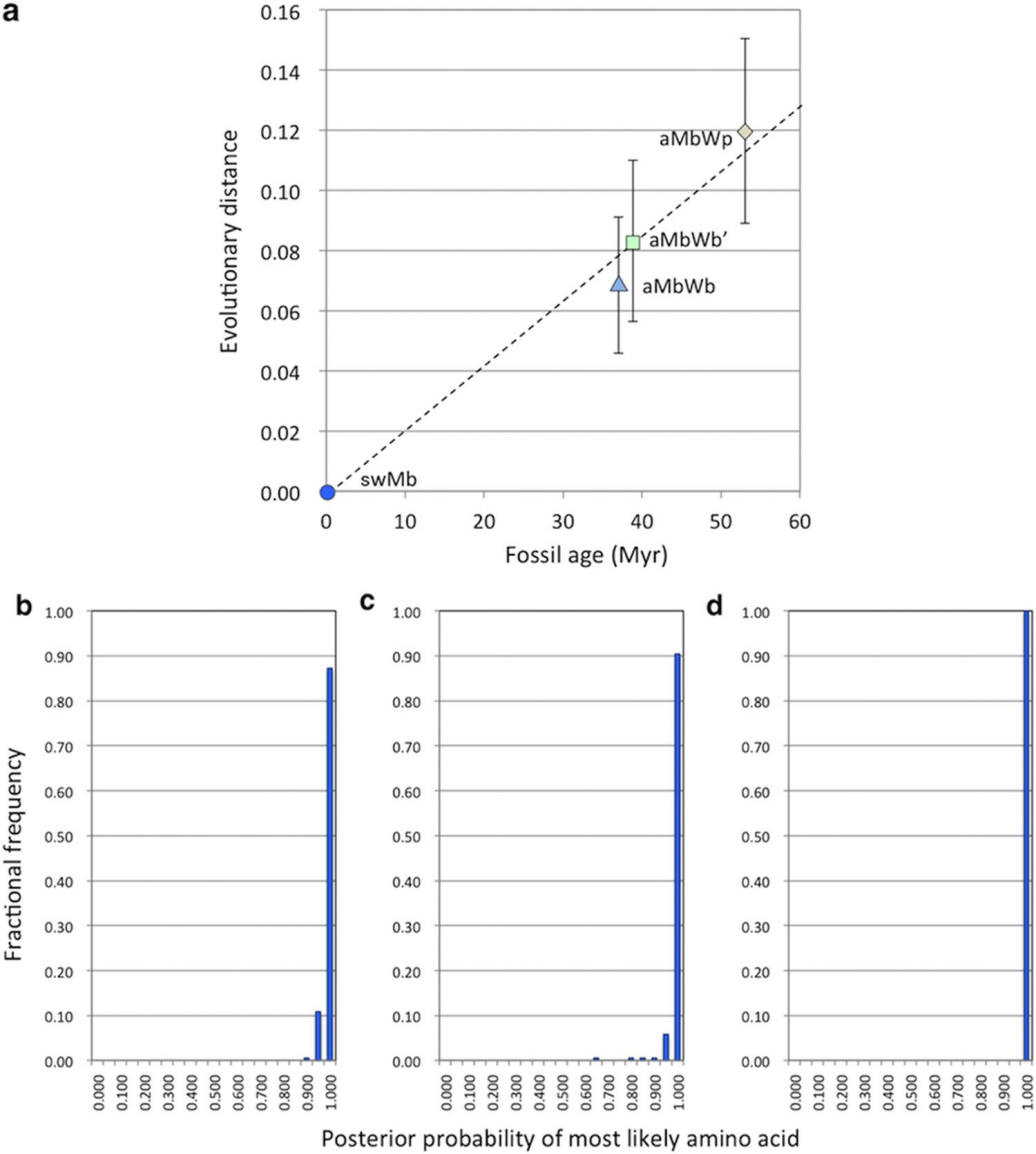
Evolutionary distance and posterior probability distribution of ancestral Mbs. (**a**) Correlation between fossil age of the whale ancestors (horizontal axis) and evolutionary distance of their inferred Mb sequences from that of extant sperm whale (vertical axis). Posterior probability distributions for the sites of aMbWp (**b**), aMbWb’ (**c**), and aMbWb (**d**) sequences. Horizontal and vertical axes show posterior probability bins and fractional frequency of inferred sites, respectively.

This highly simplified and attractive adaptation mechanism, however, still remains to be verified in a straightforward manner. A direct comparison of the proteins before and after deep-sea adaptation would provide the most conclusive and convincing evidences. In the present study, the amino-acid sequences of Mbs from extinct genera of cetaceans dated back to the Eocene epoch were inferred, based on the molecular phylogeny of Mbs, Hbs, and other closely related globins known to date, in order to elucidate the adaptation mechanisms (Figs 1 and 2, also see Fig. S2 and Table S2). The three ancestral Mbs, which are keys to understanding the molecular evolution of Mbs in diving animals, were synthesized, and their structures and chemical properties were experimentally and theoretically investigated.

## Results and Discussion

**Figure 3:**
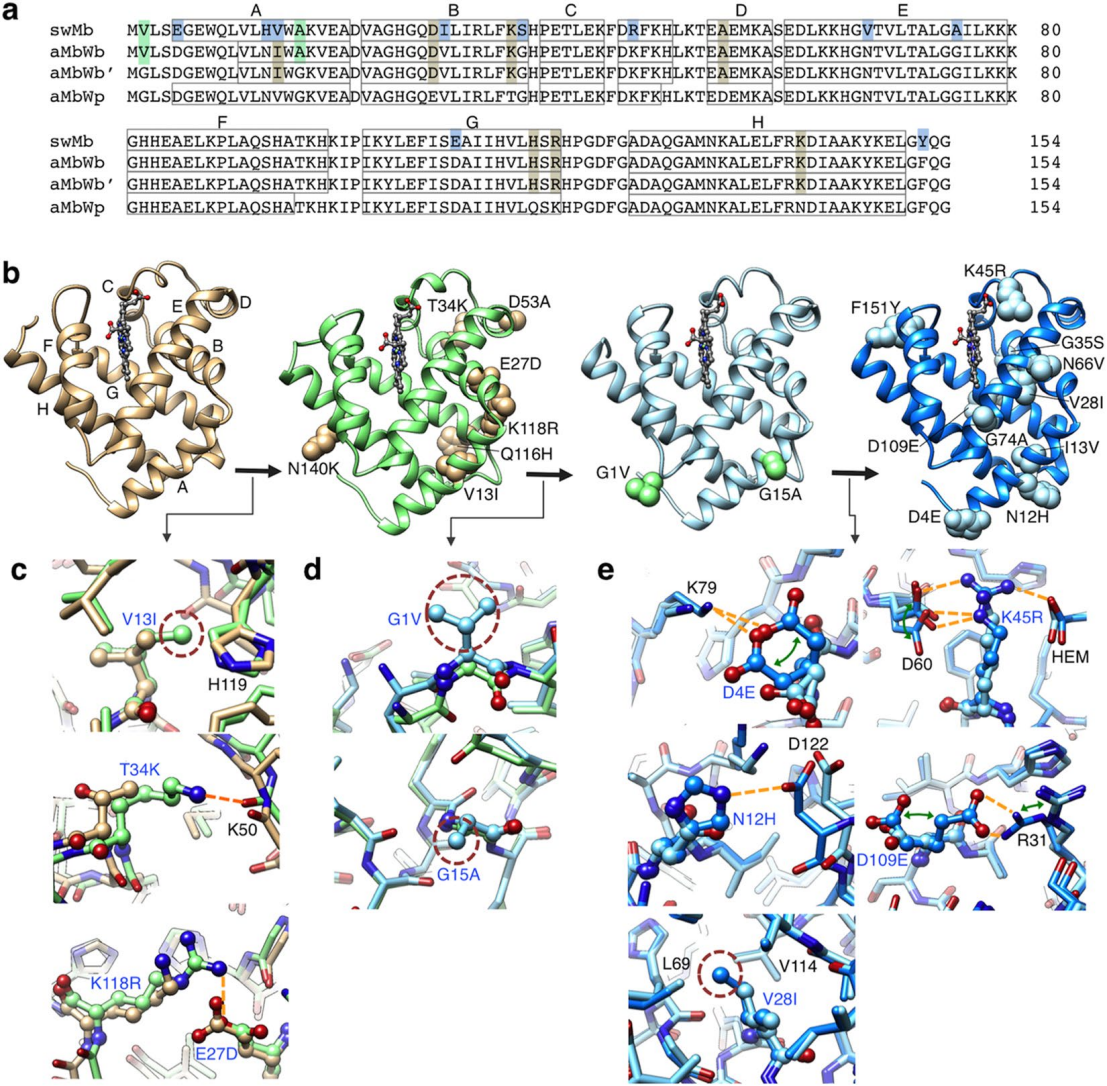
Residue replacements of whale myoglobin during the evolution from the terrestrial animal to sperm whale. (**a**) Amino-acid sequence alignment of ancestral and sperm whale Mbs. Amino acid replacements on aMbWp to aMbWb’, aMbWb’ to aMbWb, and aMbWb to swMb are meshed with light brown, light green, and light blue, respectively. The residues in the canonical helices A – H are boxed. (**b**) The replaced residues are shown on the crystal structures of aMbWp (PDB code 5YCG), aMbWb’ (5YCI and 5YCJ), aMbWb (5YCH), and swMb (5YCE). The canonical helices A – H are indicated on the structure of aMbWp.  (**c**) V13I, T34K, and K118R and E27D are replacements from aMbWp (light brown) to aMbWb’ (light green). (**d**) G1V and G15A are those from aMbWb’ to aMbWb (light blue). (**e**) D4E, V28I, N12H, K45R, and D109E are from aMbWb to swMb (blue). The electrostatic interactions/hydrogen bonds and cavity filling positions are indicted with the yellow dotted lines and red circles, respectively. The green arrows indicate alternative conformations.

The amino-acid sequences of the three synthesized ancient Mbs; namely, aMbWb, aMbWb’, and aMbWp, are compared with that of the extant sperm whale Mb (swMb) in Fig. 3a. The aMbWb and aMbWb’ proteins are the common ancestors of the toothed (Odontoceti) and baleen (Mysticeti) whales, which would be closely related to the early whale *Basilosaurus*^27^. The aMbWb and aMbWb’ are based on the two major conflicting hypotheses of cetacean evolution; namely, the monophyly versus polyphyly hypotheses for Odontoceti^28,29^, respectively, and therefore are substitutable for each other. The aMbWp is from a further common ancestor of whales and hippopotamuses, which would be the quadruped terrestrial animal *Pakicetus*^30,31^ or its closely related species. The seven residue replacements; namely, E27D, V13I, T34K, D53A, Q116H, K118R and N140K, were deduced to have occurred during the evolution from aMbWp (terrestrial ancestor) to aMbWb (aquatic ancestor). Two residues, G1V and G15A, are different between aMbWb (polyphyly ancestor) and aMbWb’ (monophyly ancestor). In aMbWb’, these residues are identical to those of aMbWp. Thus, under the currently dominating monophyly hypothesis, aMbWb’ is positioned between aMbWp and aMbWb, and therefore regarded as a pseudo-ancestor in this study (Figs 1 and S2). Furthermore, the ten residue replacements, D4E, N12H, I13V, V28I, G35S, K45R, N66V, G74A, D109E and F151Y, occurred during the evolution from aMbWb to extant swMb. Consequently, a total of 17 residue replacements were deduced during the evolution from a terrestrial ancient whale to the existing sperm whale. The relative molecular masses (*Mr*), formal net charges, and formal p*I* values were calculated from the deduced amino acid sequences (Table S3).

The three ancient Mbs and the extant swMb were synthesized in the holo-forms and purified to homogeneity (see Materials and Methods). The *E*. *coli* expression yield of swMb was higher than the ancient Mbs, and their yields increased along with the whale evolution (Fig. S3). The analyses with size-exclusion chromatography and small angle X-ray scattering (SAXS) indicated that all the Mb samples used here are almost monomeric under the broad range of Mb concentration (see below). The atomic structures of the synthesized aMbWp, aMbWb’, aMbWb, and swMb were determined by X-ray crystallography to 2.4, 1.6, 1.4, and 0.8 Å resolutions, respectively (Fig. 3b and Table S4). The main chain structures were well conserved among the ancestral and extant Mbs (Fig. S4). The net surface charges *Z*_Mb_, isoelectric points (p*I*), solvation free energies (Δ*G*_solv_), and mutational folding energy changes (ΔΔ*G*_mut_) were calculated, based on the crystal structures and the trajectories of molecular dynamics simulations (Table S3 and Fig. S5), and are discussed later along with the experimental data.

**Figure 4:**
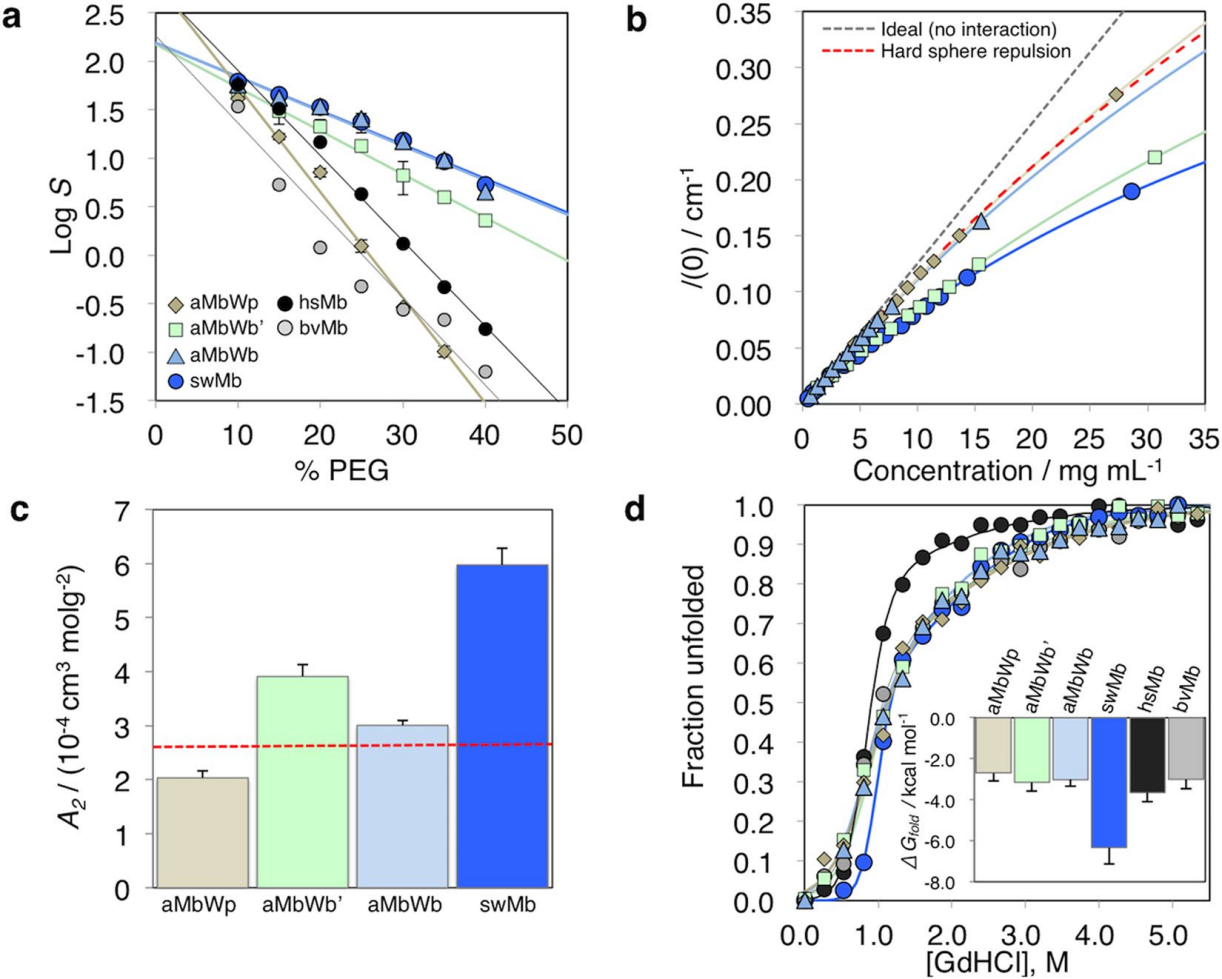
Experimental analyses of ancient and extant Mbs. The values of aMbWp, aMbWb’, aMbWb, and swMb are indicated in diamonds (light brown), squares (light green), triangles (light blue), and circles (blue). The data for horse (hsMb) and cow (bvMb) apoMbs are indicated by black and gray circles, respectively, for comparison. (**a**) Solubility dependence of holo-forms of ancient and sperm whale Mbs on the concentration of PEG-6000. Log solubility values in mg/mL (*S*) are plotted against the precipitant concentration. (**b**) Dependence of the absolute scattering intensity at *q* = 0 on the protein concentration in small angle X-ray scattering experiments (see also Fig. S6). (**c**) Estimated second virial coefficients (*A*_2_) indicating repulsive interaction between Mb molecules. *A*_2_ calculated presuming that Mb is a hard sphere is shown as a red, dashed line. (**d**) Chemical denaturation profiles of apoMbs. The unfolded fractions estimated by the CD signal intensity at 222 nm were plotted against the Gd-HCl concentration. The inset shows the *ΔG*_fold_ (kcal mol^−1^) of the proteins. The data for hsMb and bvMb are presented in black and gray, respectively, for comparison.

The synthesized Mbs were examined for their solubility, which is one of the most interesting properties of the ancestral Mbs. Generally, Mbs are highly soluble, and consequently it is difficult to obtain reproducible solubility values in normal buffer solutions, because highly concentrated protein solutions are apt to form gels and supersaturated solutions^32^. Thus, the quantitative solubility was evaluated by using polyethylene glycol (PEG) as a protein precipitant, according to the previous studies^33–35^. The solubility dependence on the PEG-6000 concentration in the protein solution was measured at room temperature, as shown in Fig. 4a. The relationship between protein solubility (*S*) and precipitant concentration can be described by a linear equation: log *S* = log *S*_0_ + β[precipitant], where log *S*_0_ is the *y*-intercept of the plot and β is the slope. *S*_0_ and β represent the solubility in water and the resistance to precipitant, respectively (Table S5). Unexpectedly, log *S*_0_ decreased during the evolution from aMbWp to aMbWb, and remained almost unchanged from aMbWb to swMb, indicating that the total solubility in pure water has not increased during evolution. This was also verified by the estimation of the solvation free energy Δ*G*_solv_ of the Mb molecules, based on a reference-modified density functional theory (Table S3)^36^. The consistent results between experimental and theoretical evaluations indicated that the observed solubility should be an intrinsic property of the Mb molecules determined by their structures. Δ*G*_solv_ increased during the evolution from aMbWp to aMbWb, whereas it minimally changed during the evolution from aMbWb to swMb, indicating that the single-molecule solubility in an aqueous solution rather decreased during the evolution. However, the resistance to the precipitant (β) significantly increased during the evolution from aMbWp to aMbWb’, slightly increased from aMbWb’ to aMbWb, and remained almost unchanged from aMbWb to swMb.

In the proposed hypothesis of Mb adaptation in diving animals, the *Z*_Mb_ increase was expected to enhance the protein solubility by preventing precipitation through positive charge repulsion among Mb molecules^11,21^, although the present results seem to be inconsistent with that hypothesis. Therefore, we measured SAXS of the Mb solutions to analyze their self-interaction potentials, in order to monitor the repulsion between Mbs (Fig. S6). The second virial coefficients (*A*_2_), which indicate either attractive (*A*_2_ < 0) or repulsive (*A*_2_ > 0) intermolecular interactions, were obtained from the analyses. The results demonstrated that the intermolecular repulsion has increased during evolution, not only from aMbWp to aMbWb but also from aMbWb to swMb (Fig. 4b,c).

Taken together, both *Z*_Mb_ and p*I* significantly increased during the evolution from aMbWp to aMbWb as previously hypothesized, whereas their increases are small during the evolution from aMbWb to swMb (Table S3 and Fig. S7). Contrary to the hypothesis, the solubility (log *S*_0_) was shown to decrease, despite the increase in *Z*_Mb_ during the early stage of the evolution (Fig. 4a and Table S5), and the molecular repulsion increased even with no obvious increase in *Z*_Mb_ during the later stage of the evolution (Fig. 4c). It is remarkable that the positive charges on the protein surface provide only a small contribution toward increasing the solubility, and even decrease it in some cases^35,37–39^. The effect of repulsion between the Mb molecules due to the acquired positive charges appears to be largely compensated by the decrease in the solubility of single molecules.

However, the increase in the parameter *β*, demonstrated by the PEG sedimentation experiments, would significantly contribute toward increasing the Mb concentration (Fig. 4a), therefore suggesting that the *Z*_Mb_ increase might be a strategy to prevent sedimentation-induced interactions with precipitant molecules other than self-aggregation of cognate molecules. The inside of living cells, including myocytes, is crowded by high concentrations of metabolites and biomolecules^40–42^; thus, those molecular crowders are potential precipitants for Mbs and could be mimicked by PEG in the sedimentation experiments. Therefore, the present results require a revision of the physiological effects of the *Z*_Mb_ increase in the Mb evolution^11,21^. In general, the effect of polymer precipitants, such as PEG, has been thought to arise from the depletion force, which depends on the volume of a protein excluding the polymer precipitants, i.e., the excluded volume^43^. However, in the present case, the excluded volumes of the extant and ancient Mbs are almost identical, and thus the enhancement of the precipitant tolerance should not be attributed to the depletion force but to the changes in protein surface properties, including *Z*_Mb_, during the evolution.

The analyses also revealed that the adaptation *via* the *Z*_Mb_-increase strategy reached a plateau at the last common ancestor of whales (aMbWb’ or aMbWb). However, not all of the offspring of the common whale ancestor have highly adapted to deep-sea environments. The maximum diving depth of Mysticeti species is 200~300 m^44^. Among the Odontoceti species, the sperm whale is an ‘elite diver’, which can dive to over 2000 m in depth, while dolphins do not dive so deep. Therefore, a considerable part of the molecular adaptation should also be observed between aMbWb’ and swMb, which suggests that the *Z*_Mb_ increase was not the sole strategy in the whale Mb evolution.

It is also hypothesized that thermodynamic stabilization might contribute to maintaining the higher Mb concentration in the myocytes. Dasmeh *et al*. computationally predicted that the folding stabilities of extant whale Mbs were higher than those of ancient whale Mbs, based on 3D modeling with their inferred amino acid sequences and extant Mb structures^23^. Olson and coworkers demonstrated that the *in vivo* expression of mammalian Mb is governed by the apoMb stability, since the rate of aggregation of unfolded apoMb is significantly higher than that of holoMb, and thus a larger fraction of folded apoMb that can immediately bind heme is the key for the high-level expression of holoMb^24^. We performed chemical denaturation experiments of apoMbs with guanidine hydrochloride (GdHCl), as shown in Fig. 4d. The denaturation profiles were analyzed by assuming the three-state transition^45–47^, and the thermodynamic parameters were estimated for the folding reactions (Table S6). The folding free energy changes from the intermediate state to the native state (Δ*G*_1_) and the total free energy changes from the unfolded to the native (Δ*G*_fold_) slightly decreased from aMbWp to aMbWb, whereas they significantly decreased from aMbWb to swMb. These results indicate that the fold stability mainly improved during the late phase of the evolution.

The stabilization mechanism was clearly observed in the Mb crystal structures. No residue insertion/deletion was anticipated during the evolution from aMbWp to swMb, and all of the changes in the molecular properties should arise from the side-chain replacements. On the crystal structures of aMbs, the residues replaced during the evolution were mostly localized on the molecular surface (Fig. 3b). The backbone structures, as well as the positions and conformations of the bound heme, did not substantially change. Most of the residues that were replaced between the ancestral and extant Mbs were suggested to increase the fold stability, and were found to have introduced additional interactions in the crystal structures (Fig. 3c–e). From aMbWp to aMbWb’, V13I filled a cavity in the hydrophobic core, T34K added a hydrogen bond, and K118R (interacting with another substitution, E27D) introduced an electrostatic interaction on the molecular surface (Fig. 3c). Although the side chains of G1V and G15A from aMbWb’ to aMbWb did not appear to be interacting with other residues, their introduction might increase the rigidity of the main chain conformation (Fig. 3d). V28I contributed to cavity filling, while D4E, N12H, K45R, and D109E enhanced the electrostatic interactions from aMbWb to swMb (Fig. 3e). Most of the additional interactions are formed by increasing the reach of the side chains. Consistently, the molecular weight (*Mr*) increased during the evolution from aMbWp to swMb. The structure stabilization with the replaced side chains was also evaluated with the structure-based computational method (Table S3 and Fig. S5). The contributions of each residue replacement to the total stability were consistent with the experimental results; *i*.*e*., V28I, G1V, V13I, K118R, and G15A were among the highest-contributing replacements to the fold stability.

**Figure 5:**
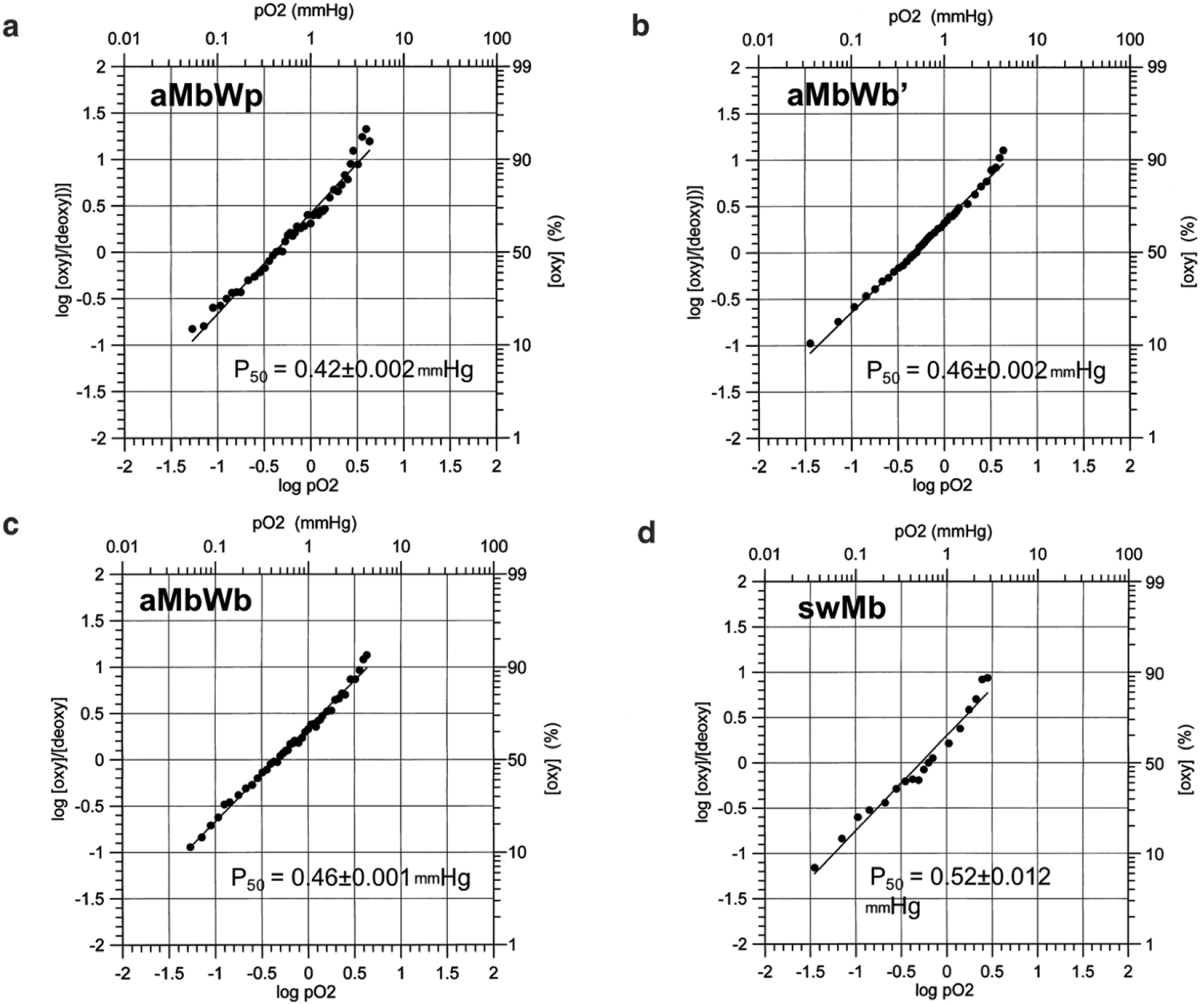
Oxygen equilibrium curves of ancient and sperm whale Mbs. The logarithmic ratios of oxy-myoglobin to deoxy-myoglobin are plotted against the logarithmic oxygen partial pressure (pO_2_) for (**a**) aMbWp, (**b**) aMbWb’, (**c**) aMbWb, and (**d**) swMb.

Finally, the major biological function of Mbs, oxygen binding, was examined. The fractional occupancy of ferrous Mb by O_2_ in a buffer solution was measured under equilibrium conditions with varied partial pressures of oxygen (*p*O_2_), and the oxygen affinity was analyzed by a Hill plot (Fig. 5). The values of *P*_50_, *p*O_2_, at which 50% of Mb are filled, were 0.42, 0.46, 0.46, and 0.52 mmHg for aMbWp, aMbWb, aMbWb’, and swMb, respectively. The slight increase in *P*_50_ values might contribute to an elevated supply of O_2_ in the deep-sea environment, since Mbs with these *P*_50_ values are saturated with O_2_ under atmospheric pressure at the sea surface, and Mbs with higher *P*_50_ values can be fully depleted of the bound O_2_ under hypoxic conditions. Two of the mutations from aMbWb to swMb, namely V28I and K45R, were located close to the heme moiety (Fig. 3e). R45 formed a salt bridge with heme-6-propionate at the O_2_ entrance, and I28 was located in the distal pocket for heme ligands. The increase in side chain volume at position 28, which eliminated the empty volume of the distal pocket as V28I mutation did, was shown to have remarkable effect in reducing ligand entry rate^48^, and might explain the slight increase in *P*_50_ value from aMbWb to swMb.

The results, however, demonstrated that the oxygen affinity of Mb was not significantly changed during the adaptation process. The *P*_50_ values for extant animals were reported previously, and indicated that deep-sea and land animal Mbs had similar O_2_-binding affinities^49^, which was consistent with the present results. This seems to be reasonable because whales adsorb O_2_ from the air on the sea surface, where *p*O_2_ condition is similar to that for land animals, and enhancement of the O_2_ affinity is not beneficial for the deep-sea adaptation. In order to increase a total supply of O_2_ from Mbs, the most reasonable strategy should be increasing the Mb concentrations in myocytes. Thus, the Mb adaptation was incarnated by the enhancements of precipitant resistance and thermodynamic stability. This is strongly supported by the values of precipitant resistance (β) and thermodynamic stability (Δ*G*_fold_) of extant land animal Mbs, which are similar to those of aMbWp, as shown in Fig. 4a,d, Tables S5 and S6 as follows.

In order to verify the significance of the observed molecular properties of ancestral Mbs to the deep-sea adaptation, the PEG sedimentation and chemical denaturation experiments were also performed for extant horse and cow Mbs, and compared with those for swMb and ancestral whale Mbs (Fig. 4a,d, Tables S5 and S6). The precipitant tolerance of these land animal Mbs were similar to the terrestrial ancestral whale Mb (aMbWp), whereas their stabilities were similar to the early ancestral whale Mbs (aMWb’ and aMbWb). These results showed that the precipitant tolerance was not improved at all, and the stability was only slightly improved during the land animal evolution, indicating the relevance of the improved stability and precipitant tolerance to the deep-sea adaptation.

**Figure 6:**
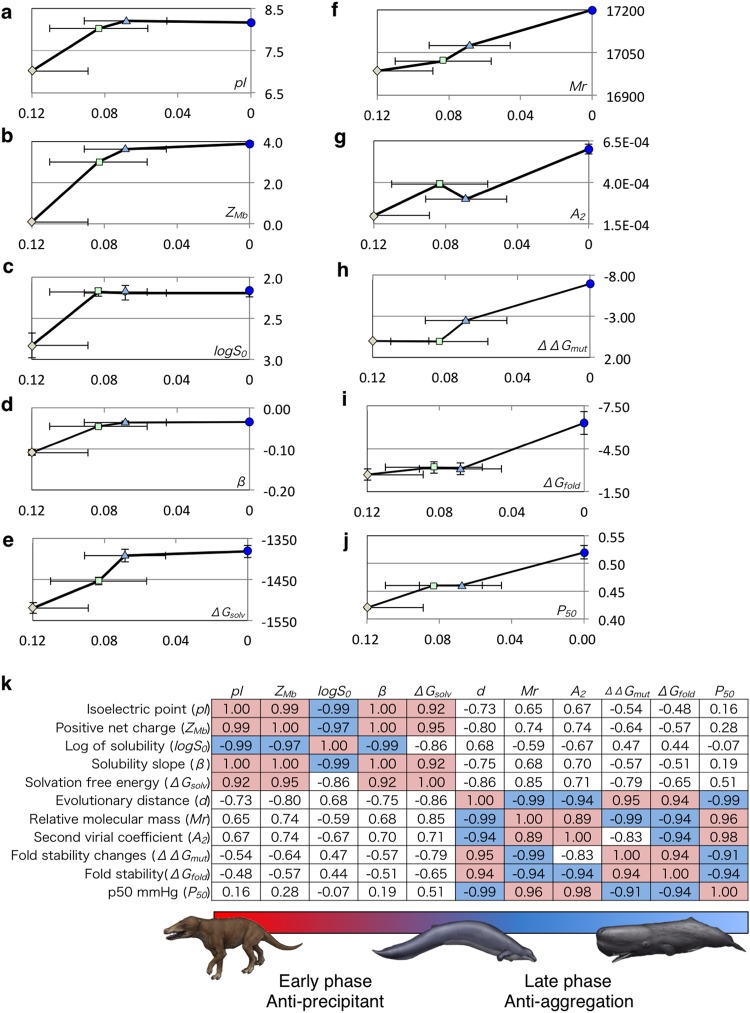
Molecular properties of ancestral and extant Mbs. The values of aMbWp, aMbWb’, aMbWb, and swMb are indicated by diamonds (light brown), squares (light green), triangles (light blue), and circles (blue). The horizontal axis shows the evolutionary distance (*d*) of each Mb sequence from that of extant swMb, and the data points are those of aMbWp, aMbWb’, aMbWb, and swMb from left to right. (**a**) Isoelectric point (p*I*), (**b**) positive net charge (*Z*_Mb_), (**c**) log of solubility (log *S*_0_), (**d**) solubility slope against precipitant (*β*), (**e**) solvation free energy (*ΔG*_solv_), (**f**) relative molecular mass (*Mr*), (**g**) second virial coefficient (*A*_2_), (**h**) mutational folding energy changes (*ΔΔG*_mut_), (**i**) folding free energy (*ΔG*_fold_), and (**j**) half-saturation oxygen pressure (*P*_50_). (**k**) Correlation coefficients between the values shown in panels (**a**–**j**) and evolutional distance. The highly positive (>0.9) and negative (<−0.9) coefficients are meshed in magenta and blue, respectively. The two clusters of molecular properties correspond to the early (from terrestrial to ancestral whale) and late (from ancestral whale to extant whale) evolutionary phases. The illustrations of animals are not covered by the CC BY license. Credit to Satoshi Kawasaki. All rights reserved, used with permission.

The theoretical and experimental results obtained in this study are summarized in Fig. 6. The present results demonstrated that the deep-sea adaptation of whale Mb should be divided into early and late phases. In the early phase, the Mb solubility in crowded intracellular conditions significantly improved with other property changes indicated in Fig. 6 to obtain the precipitant tolerance, but the single-molecule solvation free energy increased. The changes in the isoelectric point (p*I*), *Z*_Mb_, log *S*_0_, *β*, and the solvation free energy (*ΔG*_solv_) are highly correlated (Fig. 6k). The *Mr*, the second virial coefficient (*A*_2_), the mutational folding energy changes (*ΔΔG*_mut_), and the fold stability (*ΔG*_fold_) form another high-correlation cluster. These findings implied that the short-range repulsive forces between Mbs were mainly acquired during the late phase of evolution to increase the total Mb concentration.

Since the enhancement of repulsion between Mb molecules is not correlated with the *Z*_Mb_ increase, the present results require another explanation for the molecular repulsion between Mbs, which might be related to the folding stability. Although the initiation mechanism of protein aggregation is not fully understood yet, the interactions between the hydrophobic interiors of proteins, which are exposed due to partial unfolding, are considered to play a crucial role^50,51^. Thus, the folding stability would contribute toward preventing occasional aggregation upon molecular collision, and reduce the apparent attractive force between molecules. Dasmeh *et al*. demonstrated that the stabilization strategy was utilized mainly in the early phase^23^. However, as the second virial coefficient and the fold stability showed higher correlations with the evolutionary distance (from extant swMb to ancestral Mbs), this strategy would not be limited to the early phase and would have been adopted in both the early and late phases. The increase of repulsive force during the late phase should be due to a decrease in attractive interaction between Mb molecules at a contact distance, which is caused by the stability increase and prevents the aggregation.

Interestingly, the increase in molecular weight (*Mr*) also correlated with the second virial coefficient and the folding stability. Actually, the Mb structures revealed that most of the additional interactions are formed by replacing amino acids with larger ones. Another possible strategy that could correlate the *Mr* increase to the molecular repulsion might be the ‘surface entropy increment’. Surface entropy reduction engineering is used to crystallize proteins, or to improve the quality of crystals, by replacing flexible amino acids on the protein surface, such as arginine or lysine, with amino acids with smaller side chains, such as alanine^52–54^. It is possible that the acquired positively charged residues, along with the others replaced during evolution, introduced an opposite effect that prevents the self-association of Mbs by increasing the flexibility of the residues on the molecular surface. Consistent with this hypothesis, some of the replaced side chains that were involved in the acquired interactions; namely, D4E (interacting with K79), K45R (interacting with D60), and D109E (interacting with R31), from aMbWb to swMb were found to adopt alternative conformations (Fig. 3e).

In conclusion, a total of 17 residues were replaced on or near the protein surface of Mb, during the evolution from the terrestrial ancestor to deep-diving extant whales through the intermediate ancestor. The time range of the early phase of evolution from the terrestrial (aMbWp) to the intermediate (aMbWb or aMbWb’) is assumed to be ~10 M years, from the early Eocene to the middle Eocene, and that of the late phase from the intermediate to the sperm whale (swMb) is estimated to be approximately 40 M years from the middle Eocene to the present. Thus, the *Z*_Mb_ and the precipitant tolerance (β) had evolved first and rapidly by the nine residue replacements, whereas the repulsive interaction had evolved subsequently and rather slowly by the ten residue replacements. The correlation between *Z*_Mb_ and β is evident, but still it is not clear whether the *Z*_Mb_ increase alone could sufficiently explain the β increase or not. The physicochemical mechanism for the β enhancement is an important subject to be tackled in the future.

Resurrections of ancient proteins enable the investigation into a process of protein evolution during a particular period. This is often difficult by simply comparing the proteins of existing species, because they independently accumulate both neutral and non-neutral mutations after the particular evolutionary process, which potentially disturb the analyses. In the present study, the deep-sea adaptation process of Mb was dissected into early and late phases by resurrecting ancestral whale Mbs. The present results, however, also raise the question: why was the *Z*_Mb_-increasing strategy limited to the early phase, while the repulsion strategy was continued in the late phase. One possible explanation is that the number of positive charges that could be introduced on the relatively small Mb molecule without damaging its structural integrity is limited, and it already reached the upper-limit for whale Mbs. Another possibility is that the molecular adaptations in diving-capabilities to shallow and deep-sea depths are inevitably different. The *Z*_Mb_-increasing strategy has been shown to be widely adopted among shallow-diving animals, including water shrew, beaver, and platypus^11,21^. Comparisons of the Mb adaptations in these animals with those of whales, by applying the ancient protein resurrection of the present study to their Mbs, will be interesting and required to answer this question.

## Materials and Methods

### Prediction of ancestral Mb sequences

The set of amino acid sequences, including 266 Mbs, 2,179 Hbs, and 31 other globins, was retrieved from the Genbank, Refseq, and UniProt databases with BLAST by using swMb sequence as a query^55–57^. The sequences were aligned by using ClustalW, and manually refined with the XCED program^58,59^. The topology of the phylogenetic tree was inferred with the neighbor-joining (NJ) method on the JTT matrix^60,61^, and manually refined by referring to the literatures^21,62,63^ (Fig. S2a).

The phylogeny and the alignment were used to infer the ancestral sequences with the PMAL application^64–66^ (Figs 1, S2b and S2c). The last common ancestor of whales is thought to be a species of *Basilosaurus*, dated back to ~37 Mya^27^. The extant whales are classified into the morphologically and physiologically distinct Odontoceti (toothed whales) and Mysticeti (baleen whales) classes, and the monophyly of Odontoceti has been a longstanding problem^28,29,67–70^. Therefore, the whale ancestral sequences were inferred based on both the polyphyly and the monophyly hypotheses. The corresponding ancestral sequences in the former and the latter hypotheses were called aMbWb’ and aMbWb, respectively (Fig. 1). The last four-footed land ancestor of the whales is assumed to be a *Pakicetus* species dated back to ~53 Mya, and the corresponding ancestral sequence was called aMbWp^71,72^. The correlation between fossil dates and the evolutionary distances, and the distributions of the posterior probabilities of the sites were verified for the ancestral Mbs (Fig. 2 and Table S2.).

### Protein synthesis and purification

The Mbs (aMbWp, aMbWb’, aMbWb, and swMb) were synthesized from artificial genes. *E*. *coli* strain BL21 (DE3) was transformed with the vector DNA harboring each Mb gene, and the recombinant protein was synthesized by expression. The proteins were purified from the extracts by Ni Sepharose 6 Fast Flow resin (GE Healthcare) chromatography. The His-tagged Mb was digested with thrombin, and the tag was removed by a His GraviTrap mini column (GE Healthcare). The protein was finally purified by size-exclusion chromatography. The protein identities were verified by matrix assisted laser-desorption time of flight (MALDI-TOF) mass spectrometry by using an AXIMA-CFR plus mass spectrometer (Shimadzu).

For the apoMbs, the genes were subcloned into the expression vector pRSET-C (Invitrogen), and the proteins were synthesized and harvested as mentioned above. The proteins were mainly expressed as inclusion bodies, and extracted from the insoluble fraction with 6 M urea by centrifugation. After dialysis against 0.1% trifluoroacetic acid, the proteins were purified from the extracts by reversed phase HPLC with an Inertsil WP300 C18 column (GL Science). The proteins were finally purified by size-exclusion chromatography.

### Oxygen binding analyses

The purified ancestral Mbs (aMbWp, aMbWb’, aMbWb) and the recombinant sperm whale Mb (swMb) were prepared in 0.1 M sodium phosphate buffer (pH 7.0), containing the mixture of reducing enzymes and substrates of the heme reduction system, which changed the proteins’ heme state from ferric (met-form) to ferrous (oxy-form) overnight at 25 °C^73^. The heme concentrations were estimated from the absorption spectra between 700 and 400 nm, using the ratio of absorbance at 409 nm vs. 280 nm for the met-form, and the absorbances at 542 and 581 nm of the molar extinction coefficients of horse Mb for the oxy-form^74^.

The deoxy Mbs (60 µM) were obtained by degasification of oxyMb samples placed in a tonometer (230 mL volume) attached to a glass cell and sealed with a high pressure rubber cap, until the absorbance at 562 nm became stable. Then, small amounts of air were accurately inserted into the tonometer by graduation from 50 to 500 µL, and gently mixed for equilibration for 30 s. The changes in spectra between 700 and 400 nm and the absorbances at 562 nm of the deoxy Mbs were monitored with an Agilent 8453 ultraviolet-visible spectrophotometer at 25 °C until convergence. The Hill plots of oxygen equilibrium curves (OEC) of the Mbs were obtained by plotting the logarithmic oxygen saturation ratio log([oxy]/[deoxy]) against logarithmic oxygen partial pressure pO_2_. The *P*_50_ values of the Mbs were calculated with the cumulative standard deviation of the pO_2_, due to the use of the tonometer (Fig. 5).

### Crystal structure analyses

The crystal structures of the Mbs were determined by X-ray crystallography. The swMb and aMbWb crystals were grown by the batch method in a 76% saturated ammonium sulfate solution containing 6.5% (*w*/*v*) swMb or 2% (*w*/*v*) aMbWb^75^. The crystals of aMbWb’ and aMbWp were obtained by the hanging drop vapor diffusion method, under initial conditions using 0.1 M MIB buffer (pH 9.0) containing 25% (*w*/*v*) PEG1500 for a reservoir for aMbWb’, and 3.5 M ammonium sulfate solution for aMbWp. The hanging drops were prepared by mixing the 2 μL of the reservoir solution and 2 μL of the 2% (*w*/*v*) protein solution.

X-ray diffraction data were collected under cryogenic conditions, with a CCD detector Quantum315 (ADSC) at BL38B1 or MX225 (Rayonix) at BL26B2 in SPring-8 (Hyogo, Japan). The diffraction images were processed with the MOSFLM program^76,77^. The crystal structures were solved by the molecular replacement method, using the Phaser-MR application of PHENIX or MOLREP of the CCP4 suites^78,79^, and refined by using COOT and the phenix.refine application of PHENIX^78,80^ (Table S4). The atomic coordinates and structure factors of aMbWp, aMbWb’, imidazole-ligated aMbWb’, aMbWb, and swMb have been deposited in the Protein Data Bank, with the accession codes 5YCG, 5YCI, 5YCJ, 5YCH, and 5YCE, respectively. The molecular graphics were prepared with CHIMERA^81^.

### Molecular dynamics simulations

The molecular dynamics (MD) simulations of the Mbs were performed with the AMBER12 package with the force field parameter for an O_2_ ligand heme, along with the force field ff99SB for proteins^82–84^. The Mb crystal structures determined in this study and the same structures excluding the heme moieties were used as the starting structures for the holo and apo simulations, respectively. The solvent was explicitly considered with a truncated octahedral box of a TIP3P water model equilibrated at 298 K, with periodic boundary conditions based on the particle-mesh Ewald method^82,85^. A ligand O_2_ molecule, which was absent from the crystal structures, was included in the system to make it compatible with the force field parameters used for the heme. The His residues 24, 81 and 93 in the Nδ1 (HID form), 12, 48, 64, 82, 97, 113, 116 and 119 in the Nε2 (HIE form), and 36 in the Nδ1 and Nε2 (positively charged, HIP form) were protonated. Na^+^ or Cl^−^ ions were added to obtain a neutral simulation system.

The energy minimization was first performed for 1,000 cycles by restraining the Mb heavy atoms to the original positions, which was followed by additional 2,500 cycles without restraint. Then heating from 0 to 298 K was performed for 20-ps by using the heat bath coupling algorithm by constraining the atom positions^86^. The system was then subjected to an 80-ps NTP ensemble MD calculation with the same restraints at constant temperature (298 K) and constant pressure (1 bar). After releasing all of the restraints, a 60-ns NTP ensemble MD calculation with the same controls was performed, and the last 50-ns trajectories sampled every 5 ps were used for the data analyses. The radius of gyration, the root mean square deviation (RMSD) from the starting structure, the number of hydrogen bonds between Mb and water, and the conformational energy were calculated by using the tools in the AMBER package.

### Solvation free energy calculation

The solvation free energy (SFE) calculations for the Mbs were performed with a reference-modified density functional theory (RMDFT)^36,87,88^. The site-density distribution functions of water around the Mbs were calculated by using the three-dimensional reference-interaction-site-model (3D-RISM) integral equation with the Kovalenko–Hirata (KH) closure^89^. The site-site direct correlation functions for bulk water and for the reference hard-sphere fluid were calculated using the one-dimensional (1D)-RISM integral equation with the KH closure^89^ and the effective-density approximation (EDA)^90^, respectively. For the 1D-RISM and EDA calculations, 0.00125 Å and 32,768 were employed as the grid spacing and the number of grids, respectively. The number density of water and the temperature were 0.033329 molecule/Å^3^ and 298 K, respectively. The 3D-RISM integral equations were solved for a grid of 256^3^ points in an 80 Å^3^ cubic cell, using graphics processing units (GPUs)^91^. The SFE calculation was performed for 5,000 conformations of each Mb from the MD simulations based on the equation:

1 $${\rm{\Delta }}{G}_{slov}=\langle {\rm{\Delta }}{G}_{i}\rangle -{k}_{B}T\,\mathrm{ln}\langle \exp [-(\Delta {G}_{i}-\langle \Delta {G}_{i}\rangle )/{k}_{B}T]\rangle ,$$

where $\langle \,\rangle$ indicates the ensemble average over the conformations, $\Delta {G}_{i}$ is the SFE for each conformation,$\,{k}_{B}$ is the Boltzmann constant, and $T$ is the temperature. The first term in Eq. (1) provides the simple average of $\Delta {G}_{i}$ and the second term yields the fluctuation effect on $\Delta {G}_{slov}$ due to the conformation fluctuation.

### PEG sedimentation analyses

The dependence of the Mb solubility on a precipitating agent was measured with PEG-6000. The 1.8~4.6 mM purified holo-Mb solutions in 100 mM HEPES-NaOH and 10~40% PEG-6000 were prepared. The solutions were incubated at approximately 25 °C for 2 hours, and were then centrifuged to remove the precipitates. The Mb concentration (measured solubility *S*) in the supernatant was determined with the Nano Drop spectrometer by using an *ε*_409nm_ of 157000 M^−1^ cm^−1^ ^92^. The relationship between the protein solubility and the precipitant concentration was analyzed by assuming

2 $$\mathrm{Log}\,S=\,\mathrm{Log}\,{S}_{0}+{\rm{\beta }}[{\rm{precipitant}}]$$

where *S*_0_ and β are the solubility in the absence of precipitant and the dependence of the solubility on the precipitant concentration (Fig. 4a)^35^.

### Small Angle X-ray Scattering

The small angle X-ray scattering (SAXS) experiments were performed at the beam line BL-10C, in the Photon Factory (PF) of the High Energy Accelerator Research Organization (KEK), Tsukuba, Japan. The purified Mb solutions were dialyzed against a 2 mM HEPES–NaOH buffer solution (pH 6.8) at 4 °C for one day. The dialyzed Mb solutions were concentrated to ~3–5 mM, and then centrifuged to remove the precipitate. The Mb solutions were diluted to the desired concentrations at a pH of 6.9 ± 0.1, and irradiated with X-ray wavelength *λ* = 0.15 nm (camera length of 1 m) for 2 s in a cell with quartz windows using a sample-flow system (~14.5 μL/min) at 20 ± 0.1 °C. X-ray intensities were recorded by a PILATUS3 2 M detector (DECTRIS Ltd., Switzerland). A total of 30 images were collected for each condition, and the circular 1D averaging of the images was performed with the program *Nika*^93^.

The scattering parameter *q* = |***q***| = 4πsin*θ*/*λ*, where ***q*** is the scattering vector and 2*θ* is the X-ray scattering angle, available in this experiment was 0.01–0.55 Å^−1^. The scattering intensity was corrected by the intensity of the incident light and the transmittance of the X-rays. The absolute scattering intensity of the protein (*I*(*q*)) was determined as

3 $${\rm{I}}(q)=[{I}_{S}(q)-(1-{c}_{p}v){I}_{B}(q)]/f$$

where *c*_p_ is the protein concentration (g/cm^3^), *v* is the specific volume of the solute (0.7425 cm^3^/g), and *f* is the correction factor to convert the observed intensity in arbitrary units to the absolute intensity in units of cm^−1^, respectively.

The absolute scattering intensity of the protein (*I*(*q*)) was extrapolated to the absolute scattering intensity *I*(0) at *q* = 0. The *I*(0) is related to the second virial coefficient *A*_2_ as

4 $$I(0)=kM{c}_{p}/(1+2{A}_{2}M{c}_{p}),$$

where *M* is the molecular weight of the protein and the *k* value is equal to *v*^2^(*ρ*_m_ − *ρ*_solv_)^2^/*N*_A_. *N*_A_ is Avogadro’s number, and *ρ*_m_ − *ρ*_solv_ is the electron density difference between the protein and the solvent (2.8 × 10^10^ cm^−2^, typically)^94,95^.

### Folding stability analyses

The stability of Mb was determined by chemical denaturation experiments with guanidine hydrochloride (Gd-HCl) by monitoring the circular-dichroism (CD) signal intensity at 222 nm for 5 μM proteins in a buffer solution containing 50 mM HEPES-NaOH (pH 7). The denaturation data were analyzed using a theoretical curve derived from the three state transition model^45^:

5 $${\rm{F}}\,\mathop{\Longleftrightarrow }\limits^{{K}_{1}}\,{\rm{I}}\,\mathop{\Longleftrightarrow }\limits^{{K}_{2}}\,{\rm{U}}$$

where F, I and U represent the folded, intermediate and unfolded states, respectively; and *K*_1_ = [F]/[I] and *K*_2_ = [I]/[U] are the equilibrium constants of F ⇔ I and I ⇔ U, respectively.

*K*_1_ and *K*_2_ give Δ*G*_1_ and Δ*G*_2_, the free energy of the folded state relative to the intermediate and that of the intermediate relative to the unfolded state, respectively, as follows:

6 $${\rm{\Delta }}{G}_{1}={G}_{F}-{G}_{I}=-\,RT\,\mathrm{ln}\,{K}_{1}={\rm{\Delta }}G{^\circ }_{1}+{m}_{1}x$$

7 $${\rm{\Delta }}{G}_{2}={G}_{I}-{G}_{U}=-\,RT\,\mathrm{ln}\,{K}_{2}={\rm{\Delta }}G{^\circ }_{2}+{m}_{2}x$$

where Δ*G*°_1_ and Δ*G*°_2_ are the Δ*G*_1_ and Δ*G*_2_ values in the absence of denaturant, respectively, and *m*_1_ and *m*_2_ are the dependences of Δ*G*_1_ and Δ*G*_2_ on *x*, the denaturant concentration, respectively. From these relationships, the following formulas were obtained:

8 $$\alpha =1/\{1+\exp \,A+\exp (-B)\}$$

9 $$\beta =\exp (-B)/\{1+\exp \,A+\exp (-B)\}$$

where *α* and *β* are the fractions of the intermediate and the unfolded state, respectively, and *A* = −(Δ*G*°_1_ + *m*_1_ *x*)/*RT*; *B* = −(Δ*G*°_2_ + *m*_2_ *x*)/*RT*. Accordingly, the ratio of the helical content in the transition region per the total helical content of the folded form (*y*), is calculated as

10 $$y=1-\alpha -\beta +{\rm{\gamma }}a=({\rm{\gamma }}+\exp \,A)/\{1+\exp \,A+\exp (-B)\}$$

where γ is the ratio of the helical content of the intermediate per that of the folded state by assuming the helical content of the unfolded state to be zero^46,47^. The theoretical curves derived from Eq (10) were fitted to the observed denaturation data to obtain the thermodynamic parameters Δ*G*°_1_, Δ*G*°_2_, *m*_1_ and *m*_2_. The sum of Δ*G*_1_° and Δ*G*°_2_ (Δ*G*°_1+2_ =Δ*G*°_1_ + Δ*G*°_2_) gives the free energy changes from the unfolded state to the folded state.

### Accession Numbers

The atomic coordinates and structure factors have been deposited in the Protein Data Bank, www.wwpdb.org (PDB codes 5YCG, 5YCI, 5YCJ, 5YCH, and 5YCE).

## Electronic supplementary material

Supplementary Information
