# Supplementary material for: Tracing whale myoglobin evolution by resurrecting ancient proteins
Source: Sci Rep. 2018 Nov 15;8:16883. doi: 10.1038/s41598-018-34984-6 (PMC6237822; doi:10.1038/s41598-018-34984-6)
Supplement: Supplementary file 1 — Supplementary Information [file 41598_2018_34984_MOESM1_ESM.docx]

**SUPPORTING INFORMATION**

**Tracing whale myoglobin evolution by resurrecting ancient proteins**

**Yasuhiro Isogai ^1,*^, Hiroshi Imamura ^2^, Setsu Nakae ^3^, Tomonari Sumi ^4^, Ken-ichi**

**Takahashi ^3^, Taro Nakagawa ^3^, Antonio Tsuneshige ^5^ and Tsuyoshi Shirai ^3,*^**

^1^ Department of Pharmaceutical Engineering, Toyama Prefectural University, Imizu, Toyama 939-0398, Japan

^2^ Department of Applied Chemistry, College of Life Sciences, Ritsumeikan University, 1-1-1 Nojihigashi, Kusatsu, Shiga 525-8577, Japan

^3^ Department of Computer Bioscience, Nagahama Institute of Bio-Science and Technology, 1266 Tamura-Cho, Nagahama, Shiga 526-0829, Japan

^4^ Research Institute for Interdisciplinary Science, Okayama University, 3-1-1 Tsushima-Naka, Kita-ku, Okayama, 700-8530, Japan

^5^ Department of Frontier Bioscience and Research Center for Micro-Nano Technology, Hosei University, Koganei, Tokyo, Japan.

*****Correspondence authors. E-mail:**  yisogai@pu-toyama.ac.jp or t_shirai@nagahama-i-bio.ac.jp

**Supplementary Methods**

**Prediction of ancestral Mb sequences**

The initial set of amino acid sequences was retrieved from the Genbank, Refseq, and UniProt databases with BLAST, by using sperm whale (*Physeter catodon*) myoglobin (Mb) as the query ^1-3^. The sequences were aligned by using ClustalW, and manually refined with the XCED program ^4,5^. The partial sequences and the sequences that were too distantly related to the sperm whale Mb were discarded. As the result, the constructed alignment contained a total of 2,476 sequences, including 266 Mbs, 2,179 Hbs, and 31 other globins. The topology of the phylogenetic tree was inferred, based on the amino acid sequences of the extant proteins with the neighbor-joining (NJ) method on the JTT matrix ^6,7^. The tree topology was then manually refined so that it was coherent with the currently accepted species phylogeny, by referring to the literature ^8-10^. The schematically presented phylogenetic tree is shown in Fig. S2a.

The phylogeny and the alignment were subjected to a PAML analysis, to infer the ancestral sequences ^11^. The empirical model with the JTT matrix was used for the substitution model. No site partition was defined, and the substitution rate was uniform over the sites ^12,13^. The ancestral sequences were verified by reconstructing the molecular phylogenies, to determine if the inferred sequences were connected to the corresponding nodes with zero evolutionary distance (Figs. 1, S2b and S2c).

The extant whales are classified into the morphologically and physiologically distinct Odontoceti (toothed whales) and Mysticeti (baleen whales) classes, and the monophyly of Odontoceti has been a longstanding problem. Since the molecular phylogeny of mitochondrial genes suggested that the sperm whales (*Physeteroidea*) are closer to the baleen whales than to other Odontoceti, the phylogenetic position of sperm whales has been widely debated ^14-17^. The phylogeny constructed according to the globin amino acid sequences was relatively close to the former (sub-tree1 in Fig. S2b). However, the alternative phylogeny based on the SINE insertion pattern (sub-tree2 in Fig. S2c) was independent of the differences in the evolutionary rate or conservation bias, and therefore thought to reflect the actual species history ^18,19^. Therefore, the whale ancestral sequences were inferred for both phylogenies. The last common ancestor of whales is thought to be a species of *Basilosaurus*, dated back to ~37 Mya ^20^. The corresponding ancestral sequences in the former (containing sub-tree1; *ln*L = -107921.05) and latter (containing sub-tree2; *ln*L = -107906.07) phylogenies were called aMbWb’ (ancestral Mb of whale *Basilosaurus*) and aMbWb, respectively. The aMbWb’ and aMbWb sequences differed in only two positions, G1V and G15A (Fig. 2a). The last four-footed land ancestor of the whales is assumed to be a species of *Pakicetus* dated back to ~53 Mya, and the corresponding ancestral sequences, called aMbWp (ancestral Mb of whale in *Pakicetus*), were identical for both phylogenies ^21,22^. The fossil dates and the evolutionary distances in the molecular phylogeny were correlated for the ancestral Mbs (Fig. 2a). The distributions of the posterior probabilities of the sites in the ancestral sequences are shown in Figs. S3b – S3d and Table S2. Only two sites, D109 (posterior probability 0.652) and V21 (0.809) of aMbWb’, showed posterior probabilities lower than 0.9.

**Protein synthesis and purification**

The ancient whale Mbs were synthesized from artificial genes encoding the entire protein sequences. The DNA sequences were optimized for *E. coli*. The genes, cloned into a pUC vector, were purchased from FASMAC Co., Ltd., Japan. The genes were excised from the vector DNA with the *Nde* I and *Bam*H I restriction enzymes, and subcloned into the expression vector pET15b (Novagen), cleaved at the same restriction sites. *E. coli* strain BL21 (DE3) was transformed with the vector DNA, and the recombinant proteins were synthesized by expression, under the control of the T7 promoter in Terrific broth (TB) medium, which was induced by the addition of isopropyl-β-D-thiogalactopyranoside (IPTG) and hemin at 30 °C. The harvested cells were disrupted by intense sonication at 4 °C in a buffer solution, containing 0.2 M NaCl, 10 mM Tris-HCl (pH 8.0) and 0.5% β-mercaptoethanol. The synthesized protein was extracted from the solution by centrifugation, and purified from the extracts by immobilized metal affinity chromatography with Ni Sepharose 6 Fast Flow resin (GE Healthcare). The His-tagged Mb was digested with thrombin, and the His-tag fragment was removed by passage through a His GraviTrap mini column (GE Healthcare). The protein was finally purified by size-exclusion chromatography, in 50 mM HEPES-NaOH (pH 7.0) and 200 mM NaCl, on a Superdex 75 Increase 10/300 GL column (GE Healthcare). The resultant solution was concentrated with an Amicon Ultra-4 centrifugal unit, after dialysis against a buffer solution suitable for the next experiment. The protein identities were verified by matrix assisted laser-desorption time of flight (MALDI-TOF) mass spectrometry. The protein concentration of the met forms in an aqueous solution at pH 7 was determined spectrophotometrically, using *ε*_409_ = 157,000 M^-1^ cm^-1^.

The apoMbs were prepared as follows. The artificial genes were subcloned into the expression vector pRSET-C (Invitrogen), with the *Nde* I and *Bam*H I restriction enzymes. *E. coli* strain Tuner (DE3) was transformed with the vector DNA, and the recombinant proteins were synthesized by expression, under the control of the T7 promoter in TB medium, which was induced by the addition of IPTG at 37 °C. The harvested cells were disrupted by intense sonication at 4 °C in a buffer solution, containing 0.2 M NaCl, 10 mM Tris-HCl (pH 8.0) and 0.5% β-mercaptoethanol. The proteins were mainly expressed as inclusion bodies, and thus were extracted from the insoluble fraction with 6 M urea, 0.5 M NaCl, 1 mM EDTA, and 0.1% *n*-octyl-β-D-glucoside by centrifugation. After dialysis against 0.1% trifluoroacetic acid and centrifugation to remove the materials that remained insoluble under the acidic conditions without denaturant, the proteins were purified from the extracts by reversed phase HPLC with an Inertsil WP300 C18 column (GL Science). The proteins were finally purified by size-exclusion chromatography, in 50 mM HEPES-NaOH (pH 7.0) and 200 mM NaCl, on a Superdex 75 Increase 10/300 GL column (GE Healthcare). The resultant solution was concentrated with an Amicon Ultra-4 centrifugal unit, after dialysis against a buffer solution suitable for the next experiment. The protein concentration was determined spectrophotometrically, using *ε*_280_ = 15200 M^-1^ cm^-1^ ^23^.

The extant horse and bovine myoglobins (hsMb and bvMb) were purchased from Sigma and Worthington Biochemical Corporation, respectively. Their apo forms were prepared from the holo-proteins by the acid-butanone method as described previously ^24^.

**Spectroscopic analyses**

UV-visible absorption spectra were recorded using either a NanoDrop ND-1000 spectrophotometer (Thermo Scientific) with a 1 mm path length or a Hitachi U-3000 spectrometer and a quartz cuvette with a 1.0 cm path length. Circular dichroism (CD) spectra were recorded at 20 °C, using a JASCO J700 spectropolarimeter equipped with a JASCO electronic thermal controller PTC-348WI and a rectangular quartz cuvette with a 0.2 cm path length. The chemical denaturation of the apoMbs was measured by monitoring the CD signal intensity at 222 nm, with the same CD instrument. MALDI-TOF mass analyses were conducted with an AXIMA-CFR plus mass spectrometer (Shimadzu), using sinapinic acid as the matrix in the linear positive mode.

**Oxygen binding analyses**

The purified ancestral Mbs (aMbWp, aMbWb’, aMbWb) and the recombinant sperm whale Mb (swMb) were prepared in 0.1 M sodium phosphate buffer (pH 7.0), containing the mixture of reducing enzymes and substrates of the heme reduction system, which can change the proteins’ heme state from ferric (met-form) to ferrous (oxy-form) overnight at room temperature (25 ℃) ^25^.

The heme concentrations of the Mb solutions were estimated from the absorption spectra between 700 and 400 nm, using the ratio of absorbance at 409 nm vs. 280 nm for the met-form, and the absorbances at 542 and 581 nm of the molar extinction coefficients of horse Mb for the oxy-form, by a Agilent 8453 UV-visible spectrophotometer ^26^.

The deoxy Mbs (60 µM, 3 mL) were obtained by degasification of oxyMb samples placed in a tonometer (230 mL volume), attached to a glass cell and sealed with a high pressure rubber cap. This was accomplished by repeatedly replacing the gas phase with high purity nitrogen (99.9995% or higher), followed by equilibration with a gentle rocking motion, while avoiding bubble formation. This process was continued until the absorbance at 562 nm became stable, indicating full deoxyMb formation.

Small amounts of air were accurately inserted into the tonometer by graduation from 50 to 500 µL, using a micro gas-tight syringe, and gently mixed for equilibration for 30 seconds. The spectra between 700 and 400 nm and the absorbances at 562 nm of the deoxy Mbs were obtained with the spectrophotometer at 25 ℃. These operations were repeatedly performed and recorded during the absorption changes of 562 nm from the deoxy- to reoxy-forms of the Mbs.

Hill plots of the oxygen equilibrium curves (OEC) of the Mbs were presented with the Y axis of log([oxy]/ [deoxy]) of the logarithmic oxygen saturation ratio vs. the X axis of log pO_2_ of the logarithmic oxygen partial pressure (pO_2_), using experimental points that were carefully extrapolated in the vicinity of low pO_2_. The *P*_50_ values of the Mbs were calculated with the cumulative standard deviation of the pO_2_, due to the use of the tonometer.

**Crystal structure analyses**

The crystal structures of the extant and ancestral Mbs were determined by X-ray crystallography. The swMb crystals were grown by the batch method, in a 76% saturated ammonium sulfate solution containing 6.5% (*w*/*v*) swMb ^27^. The crystals of aMbWb were obtained under the same conditions as those for swMb containing 2% (*w*/*v*) aMbWb, by micro-seeding crushed swMb crystals. The crystals of aMbWb’ were obtained by the hanging drop vapor diffusion method, under conditions using 0.1 M MIB buffer (pH 9.0) containing 25% (*w*/*v*) PEG1500 for a 0.5 mL reservoir, and a mixture of 2 μL of reservoir solution and 2 μL of protein solution in 50 mM Tris-HCl (pH 8.0) buffer, containing 2% (*w*/*v*) aMbWb’ in the hanging drop. The aMbWb’ crystals were soaked 5 times in 0.5 mL of imidazole-depleted buffer, containing 25 mM sodium malonate dibasic monohydrate, 37.5 mM boric acid, and 25% (*w*/*v*) PEG1500 (pH 9.0), to remove the heme-liganded imidazoles. The crystals of aMbWp were obtained by the hanging drop vapor diffusion method, under conditions using a 3.5 M ammonium sulfate solution for a 0.5 mL reservoir, and a mixture of 2 μL of the reservoir solution and 2 μL of the protein solution in 50 mM Tris-HCl (pH 8.0) buffer, containing 2% (*w*/*v*) aMbWp for the hanging drop. All crystals were grown at 18 °C for a few weeks.

X-ray diffraction data were collected from loop-mounted crystals under cryogenic conditions, with a CCD detector Quantum315 (ADSC) at BL38B1 or MX225 (Rayonix) at BL26B2 in SPring-8 (Hyogo, Japan). The crystals were soaked for 10–30 s in the corresponding crystal growth buffer, containing 15% (*v*/*v*) 2-methyl-2,4-pentanediol (MPD) for cryoprotection. The diffraction images were processed with the MOSFLM program ^28,29^.

The crystal structures were solved by the molecular replacement method, using the Phaser-MR application of PHENIX or MOLREP of the CCP4 suites ^30,31^. The model refinements were conducted by using COOT and the phenix.refine application of PHENIX ^30,32^. The quality of the models was evaluated with the PROCHECK program ^33^. The crystallographic parameters, data collection and refinement statistics, and PDB codes are summarized in Table S4. The atomic coordinates and structure factors of aMbWp, aMbWb’, imidazole-ligated aMbWb’, aMbWb, and swMb have been deposited in the Protein Data Bank, with the accession codes 5YCG, 5YCI, 5YCJ, 5YCH, and 5YCE, respectively. The molecular graphics were prepared with CHIMERA ^34^.

**Molecular dynamics simulations**

The molecular dynamics (MD) simulations of the Mbs were performed with the AMBER12 package with the force field parameter for a heme (with an O_2_ ligand), along with the force field ff99SB for proteins ^35-37^. The crystal structures of the Mbs determined in this study were used as the starting structures for the holo simulations, and the same structures, excluding the heme moieties, were also used for the apo simulations.

A ligand O_2_ molecule, which was absent from the crystal structures, was included in the system for the holo simulations to make it compatible with the force field parameters used for the heme. The tautomeric states of the His residues at neutral pH were inferred, by using the function of protonate3D in the Molecular Operating Environment software (Chemical Computing Group), and were found to be consistent with the experimental pKa data ^10,38^. Accordingly, His residues 24, 81 and 93 in the Nδ1 (HID form), 12, 48, 64, 82, 97, 113, 116 and 119 in the Nε2 (HIE form), and 36 in the Nδ1 and Nε2 (positively charged, HIP form) were protonated. All other residues and both termini of the proteins were considered in their standard protonation state at pH 7.

The net charges of swMb, aMbWb’, aMbWb and aMbWp in the holo form were +1, +1, +1 and −2, and those in the apo form were +3, +3, +3 and 0, respectively. Na^+^ or Cl^−^ ions were added to obtain a neutral simulation system. The solvent was explicitly considered with a truncated octahedral box of a TIP3P water model, with periodic boundary conditions. The box size was set so that the minimal distance between the protein and the box faces was 10 Å. By exploiting the periodic boundary conditions, the particle-mesh Ewald method was used to treat the long-range electrostatic interactions ^35,39^. The solvated systems were energy-minimized, and heated to and equilibrated at 298 K.

The energy minimization was first performed for 1,000 cycles with the Mb heavy atoms restrained with a force constant of 500 kcal mol^-1^ Å^-2^, followed by 2,500 cycles without any restraint. The heating from 0 to 298 K was performed by using the heat bath coupling algorithm for temperature control with a time constant of 1 ps, in a 20-ps MD calculation at constant volume with the Mb atoms restrained with a force constant of 10 kcal mol^-1^ Å^-2^  ^40^. The system was then subjected to an 80-ps MD calculation at constant temperature (298K, controlled by Langevin dynamics with a collision frequency of 2 ps^-1^) and constant pressure (1 bar, controlled by isotropic position scaling with a relaxation time of 1 ps), with the same restraints on the Mb atoms. After releasing all of the restraints, a 60-ns MD calculation with the same controls for constant temperature and constant pressure (the NTP ensemble) was performed, and the last 50-ns trajectory (snapshot coordinates of the system, sampled every 5 ps) was used for the data analyses. The radius of gyration, the RMSD (root mean square deviation) from the starting structure, the number of hydrogen bonds between Mb and water, and the conformational energy of Mb were calculated, using the tools in the AMBER package.

**Solvation free energy calculation**

The solvation free energy (SFE) calculations for Mbs were performed, using a reference-modified density functional theory (RMDFT) that had been proposed as a precise and efficient liquid-state method ^41-43^. The validity of the SFE calculations determined by the RMDFT was previously assessed in comparison with the experimental data for a set of neutral amino acid side-chain analogues and 504 small organic molecules ^41^. The site-density distribution functions of water around the Mb were calculated, using the three-dimensional reference-interaction-site-model (3D-RISM) integral equation with the Kovalenko–Hirata (KH) closure ^44^. The site-site direct correlation functions for bulk water were also calculated, using the one-dimensional (1D)-RISM integral equation with the Kovalenko–Hirata (KH) closure ^44^. In these RISM calculations, we used the TIP3P model ^45^ with an additional Lennard-Jones parameter for each hydrogen site ($\sigma$= 0.4 Å and $\varepsilon$=0.046 kcal/mol) ^46^. The direct correlation function for the reference hard-sphere fluid, which was required to construct the solvation free energy functional, was determined using the effective-density approximation (EDA) ^47^, where 2.8750 Å was employed as the optimal diameter of the reference hard-sphere system ^43^. The number density of the water and the temperature were 0.033329 molecule/Å^3^ and 298 K, respectively. The 3D-RISM integral equations were solved for a grid of 256^3^ points in a cubic cell with a size of 80 Å^3^, using graphics processing units (GPUs) ^48^. We employed 0.00125 Å and 32,768 as the grid spacing and the number of grids, respectively, for the 1D-RISM and EDA calculations. We performed the SFE calculations for 5,000 conformations of each Mb that were generated by the MD simulations. To consider the fluctuation effects on the SFE, we applied the following equation:

$\text{Δ}G_{slov}=\left\langle\text{Δ}G_{i} \right\rangle-k_{B}T\text{ln}\left\langle\text{exp}\left[ -\left( \Delta G_{i}-\left\langle\Delta G_{i} \right\rangle\right)/{k_{B}T} \right] \right\rangle$, (1)

where $\left\langle\right\rangle$ indicates the ensemble average over the conformations, $\Delta G_{i}$ is the SFE for each conformation, $k_{B}$ is the Boltzmann constant, and $T$ is the temperature. In Eq. (1), the first term provides the simple average of $\Delta G_{i}$ and the second term yields the fluctuation effect on $\text{Δ}G_{slov}$ due to the conformation fluctuation.

**PEG sedimentation analyses**

The dependence of the Mb solubility on a precipitating agent was measured with PEG-6000. The purified holo-Mb samples were typically prepared at 9 mM in a diluted buffer solution, and were mixed with 50% PEG-6000, deionized water and 1 M HEPES-NaOH (pH 7.0), to give 1.8~4.6 mM Mb solutions in 100 mM HEPES-NaOH and 10~40 % PEG-6000, which are much higher than the protein solubility. The resultant solutions were incubated at room temperature (approximately 25 ºC) for 2 hours, and were then centrifuged to remove the precipitates. The Mb concentration in the supernatant was determined with the Nano Drop spectrometer by using an *ε*_409nm_ of 157000 M^-1^ cm^-1^ ^49^, and was regarded as the measured solubility *S* at each PEG concentration. The relationship between the protein solubility and the precipitant concentration was analyzed by assuming that

Log *S* = Log *S*_0_ + β [precipitant] (2)

where *S*_0_ and β are the solubility in the absence of precipitant and the dependence of the solubility on the precipitant concentration (see Fig. 4a) ^50^.

**Small Angle X-ray Scattering**

The small angle X-ray scattering (SAXS) experiments were performed at the beam line BL-10C, in the Photon Factory (PF) of the High Energy Accelerator Research Organization (KEK), Tsukuba, Japan. The X-ray wavelength, *λ*, was 0.15 nm, and the camera length was 1 m, calibrated by the use of a scattering pattern of silver behenate. X-ray intensities were recorded by a PILATUS3 2M detector (DECTRIS Ltd., Switzerland). The Mb solutions, purified by size exclusion chromatography, were dialyzed against a 2 mM HEPES–NaOH buffer solution (pH 6.8) at 4 °C for one day. The dialyzed Mb solutions were concentrated to ~ 3–5 mM, and then centrifuged to remove the precipitate. The Mb solutions were diluted to the desired concentrations, at a pH of 6.9 ± 0.1. The samples were measured at 20 ± 0.1 ºC in a cell with quartz windows for 60 s with a sample-flow system (~14.5 μL/min), to avoid radiation damage of the protein. In total, 30 images (one image per 2 s) were collected. The circular 1D averaging of the images was performed with the program *Nika* ^51^. The 1D data; *i.e.,* the scattering intensities, were averaged. The scattering parameter *q* is defined as *q* = |***q***| = 4πsin*θ*/*λ*, where ***q*** is the scattering vector, and 2*θ* is the scattering angle of the X-rays. The *q*-range available in the present study was 0.01–0.55 Å^-1^. The scattering intensity was corrected by the intensity of the incident light and the transmittance of the X-rays. The absolute scattering intensity of the protein (*I*(*q*)), in units of cm^–1^, was determined by subtracting the corrected scattering intensity of the buffer solution (*I*_B_(*q*)) from that of the protein sample solution (*I*_S_(*q*)), as follows:

*I*(*q*) = [*I*_S_(*q*) – (1 – *c*_p_*v*)*I*_B_(*q*)]/*f* (3)

where *c*_p_ is the protein concentration (g/cm^3^), *v* is the specific volume of the solute (cm^3^/g), and *f* is the correction factor to convert the observed intensity in arbitrary units to the absolute intensity in units of cm^-1^, respectively. A *v* value of 0.7425 cm^3^/g, which is known as a good approximation for a SAXS analysis ^52^, was used. The *f* value, which depends on the experimental setup, was determined by water scattering as the standard ^53,54^.

The protein scattering in the low-*q* region is approximated by

ln*I*(*q*) = *sq*^2^ + ln*I*(0) (4)

where *s* is the slope, which gives a radius of gyration (*R*g); *i.e*., *s* = –1/3*R*g^2^, at a diluted protein concentration according to the Guinier law ^55^. The absolute scattering intensity at *q* = 0, *I*(0), is rationalized to

*I*(0) = *kMc*_p_ / (1 + 2*A*_2_*Mc*_p_) (5)

where *k* is the constant, *M* is the molecular weight of the protein, and *A*_2_ is the second virial coefficient ^56,57^. The *k* value is equal to *v*^2^(*ρ*_m_ – *ρ*_solv_)^2^/*N*_A_, where *N*_A_ is Avogadro's number, and (*ρ*_m_ – *ρ*_solv_) is the electron density difference between the protein and the solvent (2.8 × 10^10^ cm^-2^, typically).

The value of *A*_2_ for an ideal solution is 0; *i.e.*, no interaction between the particles. The value of *A*_2_ for the repulsion between hard spheres with a radius *r*_s_ (cm) is theoretically given by 2π*N*_A_(2*r*_s_)^3^ / (3*M*^2^) ^57,58^. The parameters (17.5 kDa for *M* and 20 Å for *r*_s_, approximations for Mb) generated the hard sphere repulsion of *A*_2_ = 2.6 × 10^–4^ cm^3^ mol g^–2^.

**Folding stability analyses**

The thermodynamic stability of Mb was determined by denaturation experiments with guanidine hydrochloride (Gd-HCl), which were performed by monitoring the CD signal intensity at 222 nm with 5 μM protein in a buffer solution, containing 50 mM HEPES-NaOH (pH 7) and various concentrations of Gd-HCl (see Fig. 4d). The denaturation data were analyzed using a theoretical curve derived from the three state transition model:

*K*_1_ *K*_2_

F ⇔ I ⇔ U (6)

where F, I and U represent the folded, intermediate and unfolded states, respectively; and *K*_1_ and *K*_2_ are the equilibrium constants of F⇔I and I⇔U, respectively (*K*_1_=[F]/[I], *K*_2_=[I]/[U]) ^59^. *K*_1_ and *K*_2_ give Δ*G*_1_ and Δ*G*_2_, the free energy of the folded state relative to the intermediate and that of the intermediate relative to the unfolded state, respectively. Δ*G*_1_ and Δ*G*_2_ are assumed to depend linearly on the denaturant concentration:

Δ*G*_1_ = *G*_F_-*G*_I_ = -*RT* ln *K*_1_ = Δ*G*^o^_1_ + *m*_1_ *x* (7)

Δ*G*_2_ = *G*_I_-*G*_U_ = -*RT* ln *K*_2_ = Δ*G*^o^_2_ + *m*_2_ *x* (8)

where Δ*G*^o^_1_ and Δ*G*^o^_2_ are the Δ*G*_1_ and Δ*G*_2_ values in the absence of denaturant, respectively, and *m*_1_ and *m*_2_ are the dependences of Δ*G*_1_ and Δ*G*_2_ on *x*, the denaturant concentration, respectively. From these relationships, the following formulas were obtained:

*α* = 1 / {1 + exp *A* + exp (-*B*)} (9)

*β* = exp (-*B*) / {1 + exp *A* + exp (-*B*)} (10)

where *α* and *β* are the fractions of the intermediate and the unfolded state, respectively, and *A*=-(Δ*G*^o^_1_+*m*_1_ *x*)/*RT*; *B*=-(Δ*G*^o^_2_+*m*_2_ *x*)/*RT*. Accordingly, *y*, the ratio of the helical content in the transition region per the total helical content of the folded form, is calculated as

*y* = 1 - *α* - *β* + *γα* = (γ + exp *A*)/{1 + exp *A* + exp (-*B*)} (11)

where γ is the ratio of the helical content of the intermediate per that of the folded state, and the helical content of the unfolded state is assumed to be zero ^60,61^. The theoretical curves derived from Formula 11 were fitted to the denaturation data, to obtain the thermodynamic parameters Δ*G*_1_^o^, Δ*G*_2_^o^, *m*_1_ and *m*_2_. The sum of Δ*G*_1_^o^ and Δ*G*_2_^o^ (Δ*G*_1+2_^o^ =Δ*G*_1_^o^+ Δ*G*_2_^o^) gives the free energy changes from the unfolded state to the folded state in the absence of denaturant, and measures the stability of the folded state against the unfolded state, regardless of the stability of the intermediate.

**Supplementary Data**

**Fig. S1.**  Skeletal muscle Mb contents in aquatic vs. terrestrial animals. The data were taken from Table S1.


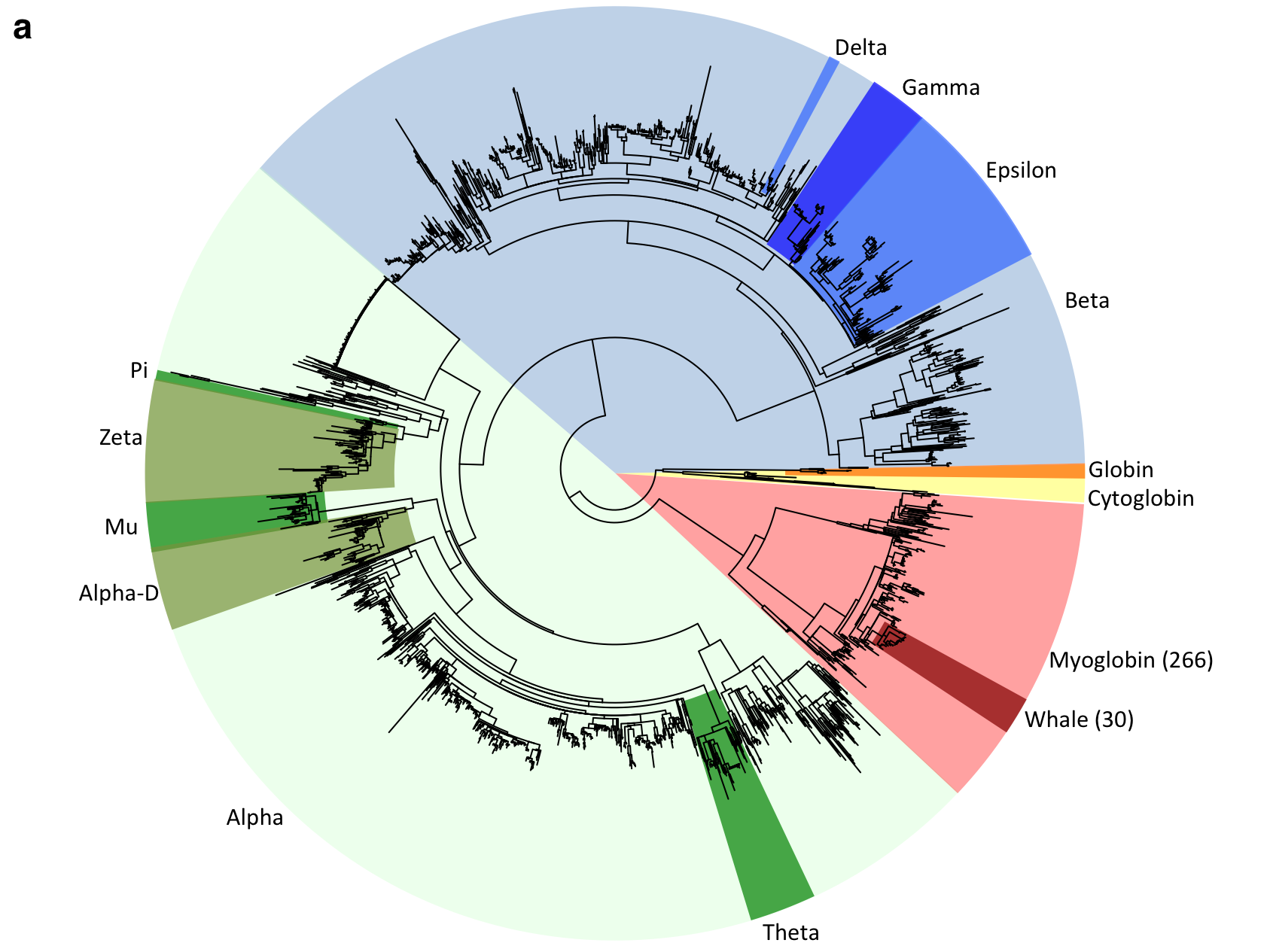


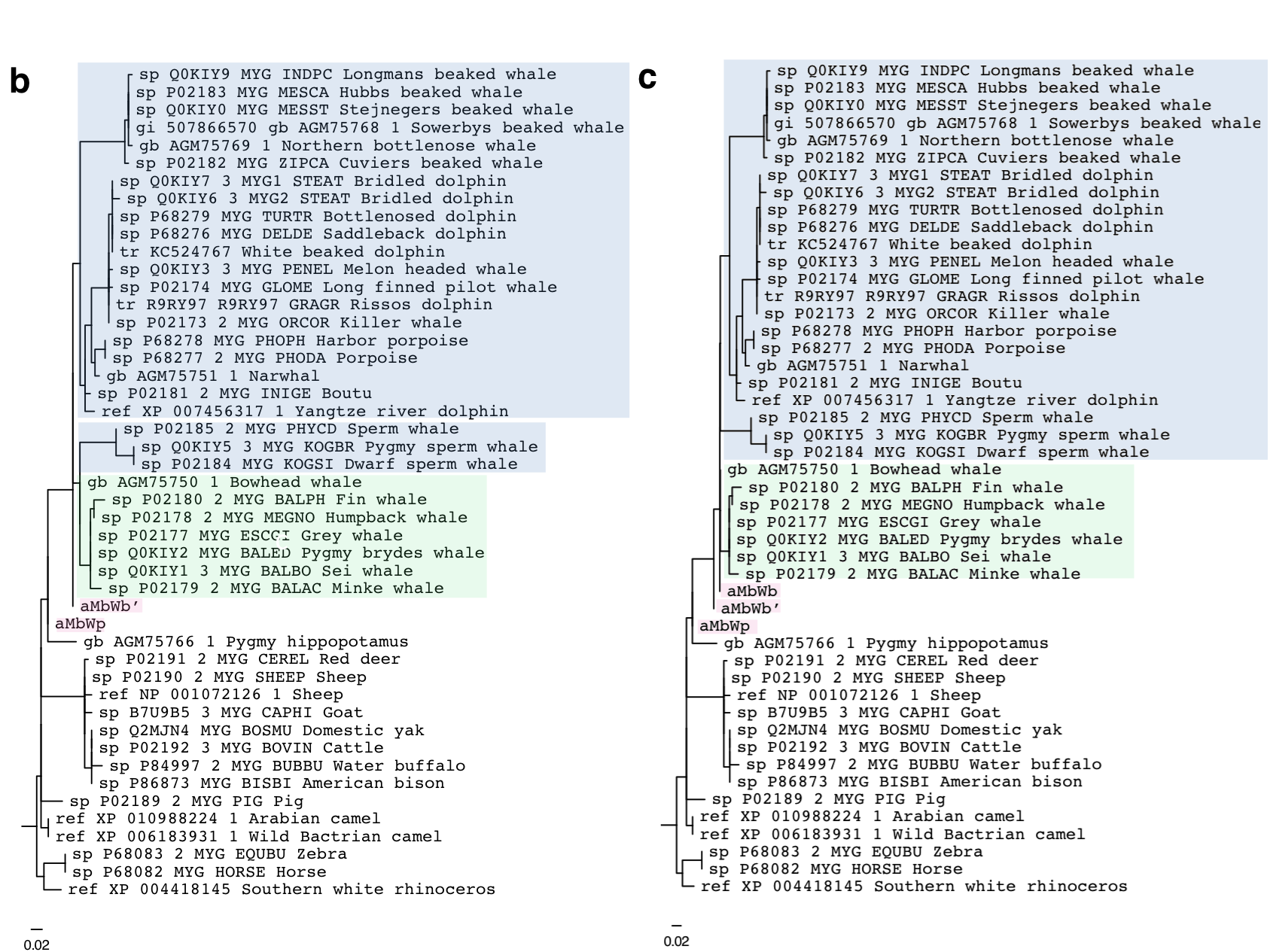


Fig. S2. Molecular phylogeny of Mb, Hb, and other globins. (a) The presented radial tree contains 266 Mbs, 2,179 Hbs, and 31 other globins. (b) Sub-tree1 consists of Mbs of whales and related land animals based on the paraphylic hypothesis for Odontoceti. (c) An alternative sub-tree2 based on the monophyly hypothesis for Odontoceti. The extant Mbs in both sub-trees are identified with data source (gb, sp, tr, and ref for Genbank, UniProt, tremble, and Refseq), accession code, species or common name. Odontoceti, Mysticeti, and ancestral Mbs are meshed in light blue, light green, and light pink, respectively.


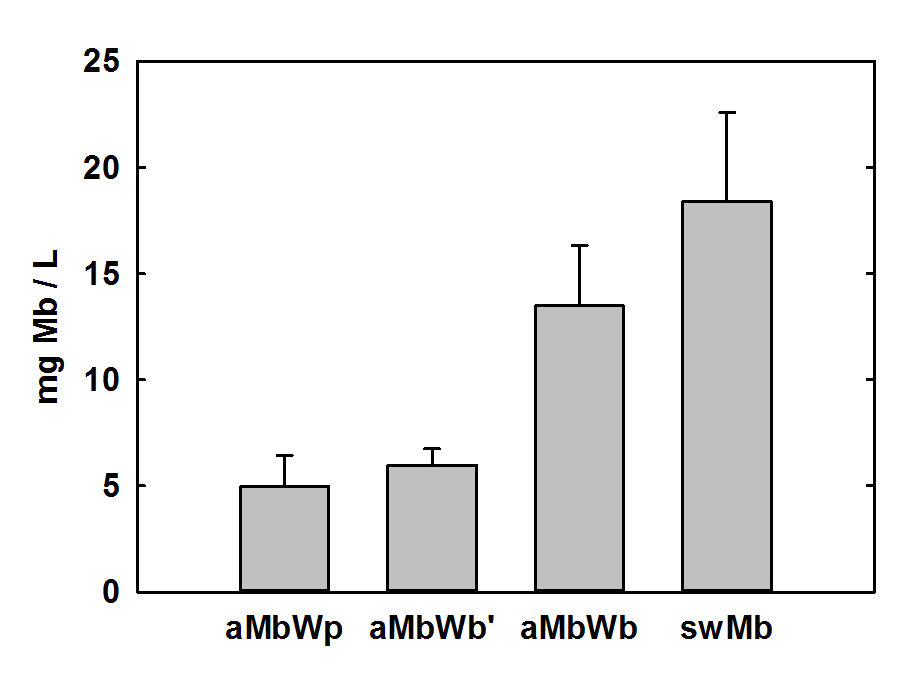


Fig. S3. *E. coli* expression yield of holoMbs into the transformant lysate. 20 ml-LB mediums containing 0.1 mg/ml ampicillin (LB Amp) were inoculated with single *E. coli* colonies containing the recombinant pET 21a expression vector, and were incubated overnight at 37 ℃. Each culture was transferred into a 500 ml-TB Amp solution, and cultivated for 2 hours at 37 ℃. The gene expression was induced by addition of IPTG at a final concentration of 1 mM, and the solution was further cultivated for 5 hours at 37 ℃. These cells were collected by centrifugation, washed with a buffer solution containing 50 mM HEPES-NaOH (pH7.0) and 200 mM NaCl (HN buffer), re-suspended in 10 mL HN buffer, and were disrupted by sonication. The insoluble materials were removed by high-speed centrifugation and the resultant supernatant solutions were assayed spectroscopically. The holo Mb contents were determined by the Soret peak with an absorption coefficient of 128000 M^-1^ cm^-1^ at 418 nm for the oxygenated forms ^23^.

**a**

**b**

**Fig. S4.** Superimposition of ancient and sperm whale Mbs. (**a**) The structures were superimposed to minimize the RMSD of each heme atom. (**b**) RMSD of C_α_ atoms between the superimposed main-chain structures. The differences of each Mb structure to the most ancient aMbWp did not increase during the evolution.

**
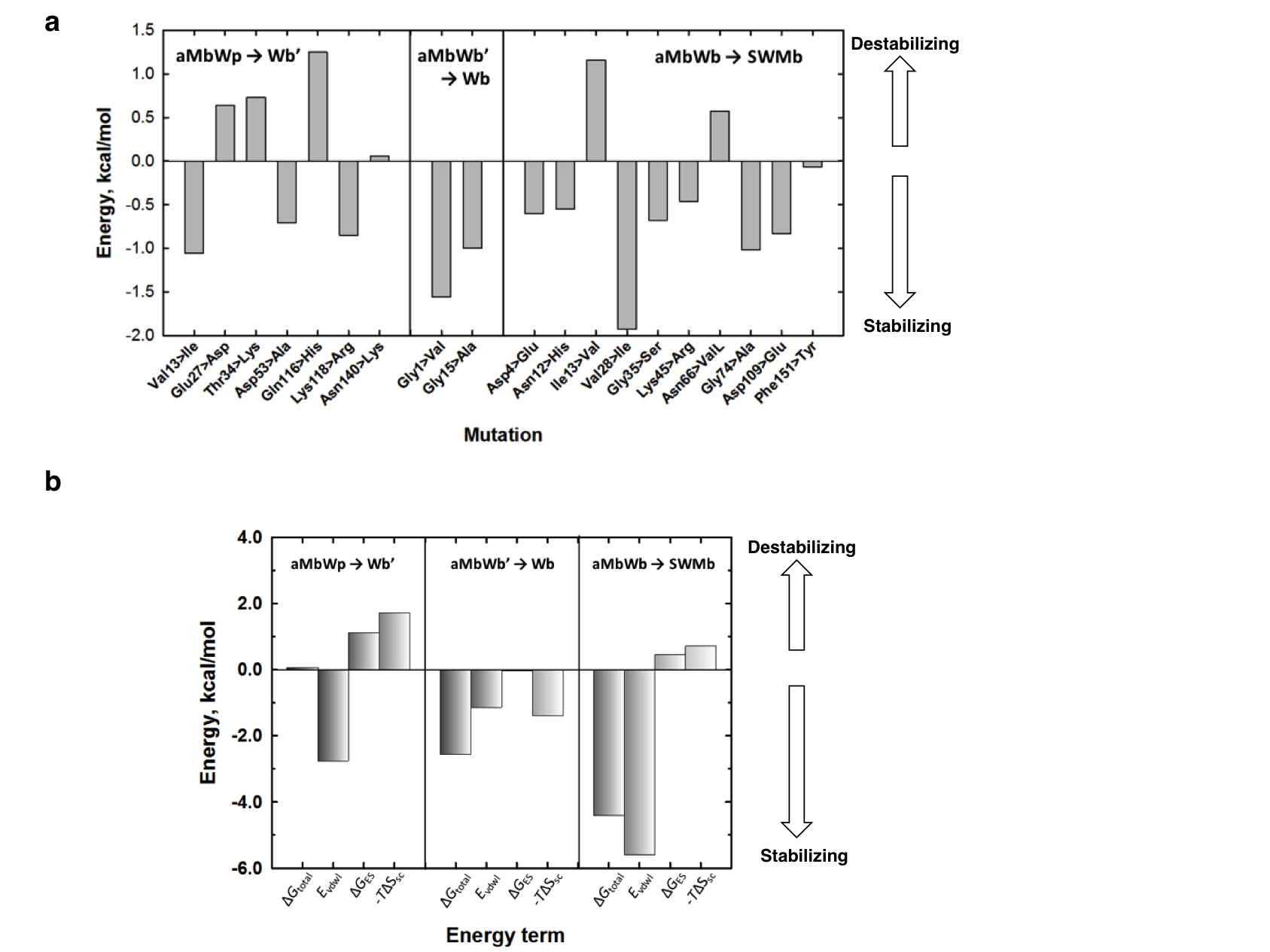
**

**Fig. S5.** Theoretical analyses of fold stability based on the X-ray structures. (**a**) Mutational energy changes (ΔΔ*G*_mut_) by the residue substitution during the evolution were evaluated. The total mutation energy changes are listed in Table S3. (**b**) Contributions of the energy terms to the total energies.

**
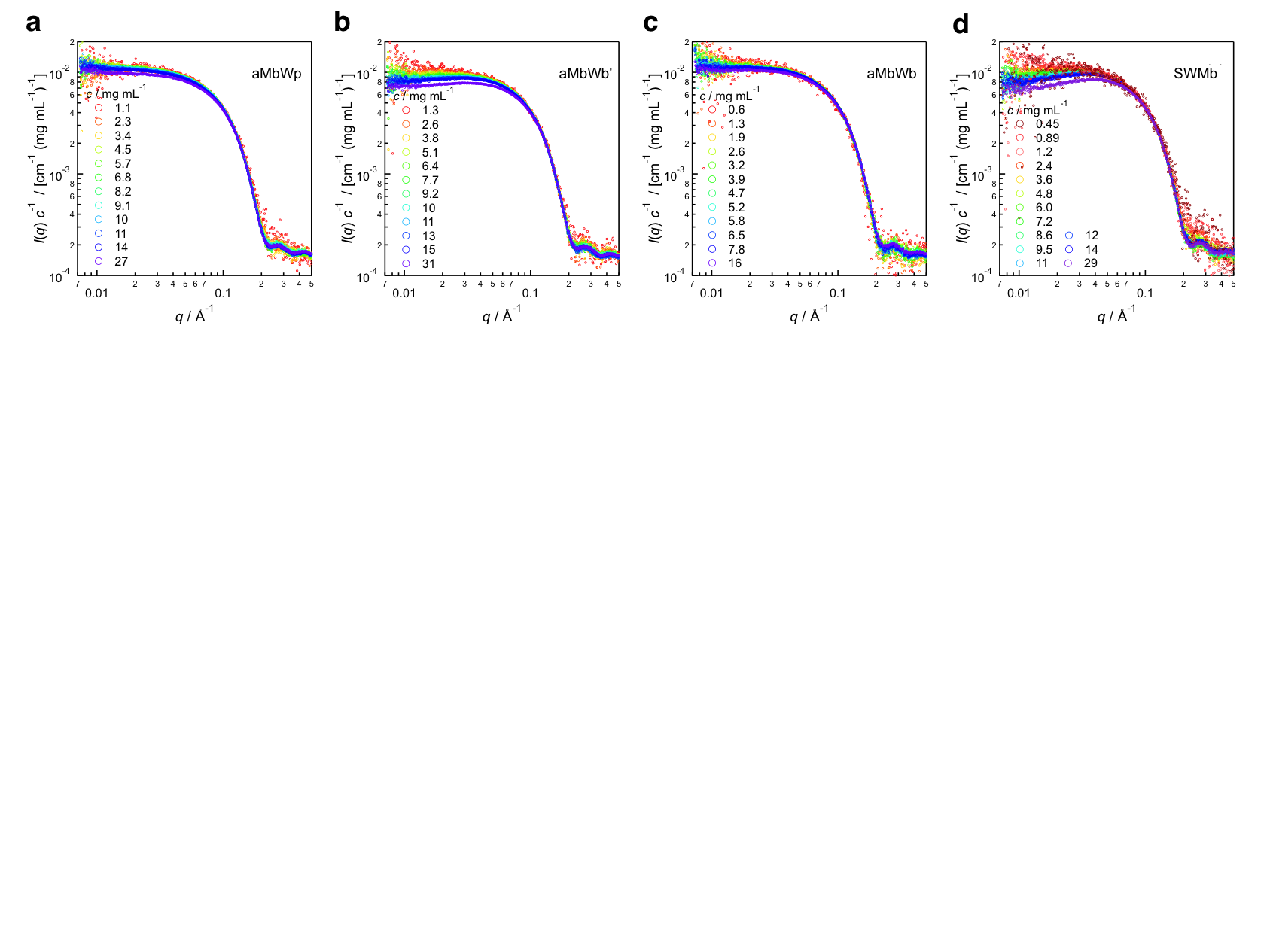
**

**Fig. S6.** Dependence of SAXS profiles of ancient and sperm whale Mbs on the protein concentration. The scattering intensities *I*(*q*) from Mb solutions are plotted against the scattering vector length *q* for aMbWp (**a**), aMbWb’ (**b**), aMbWb (**c**), and swMb (**d**). The protein concentrations are shown in color gradient from red (low concentration) to violet (high concentration) as shown in the inset of each plot.

**Fig. S7.**  Electrostatic properties of ancient and sperm whale Mbs. (**a**) pH dependence of *Z*_Mb_. (**b**) Native PAGE analysis with a 7.5% polyacrylamide gel at pH 6.8.  The gel image was not cropped from different parts of the same gel, nor from different gels.

**Table S1**. **Mb contents of muscle tissue of diving and land animals^a^**

| Species | Mb concn.  g protein/kg tissue (mM) | References |
| --- | --- | --- |
| DIVING SPECIES |  |  |
| CETACEA  Large cetaceans |  |  |
| *Balaenoptera physalus* | 24 (1.4) | Noren & Williams (2000)^62^ |
| *Balaena mysticetus* | 35 (2.1) | Noren & Williams (2000) ^62^ |
| *Balaenoptera borealis* | 9 (0.5) | Tawara (1950) ^63^ |
| *Physeter catodon* | 56.7 (3.3) | Lockyer (1976) ^64^ |
| *Hyperoodon rostratus* | 63.4 (3.7) | Scholander (1940) ^65^ |
| *Ziphius cavirostris* | 43 (2.5) | Noren & Williams (2000) ^62^ |
| Small cetaceans |  |  |
| *Pseudorca crassidens* | 63 (3.7) | Harrison & Davis (1998) ^66^ |
| *Monodon monoceros* | 79 (4.6) | Noren & Williams (2000) ^62^ |
| *Kogia breviceps* | 43 (2.5) | Noren & Williams (2000) ^62^ |
| *Tursiops truncatus* | 32.5 (1.9) | Blessing & Hartschen-Niemeyer (1969) ^67^ |
| *Delphinus delphis* | 36 (2.1) | Noren & Williams (2000) ^62^ |
| *Phocaena phocaena* | 41 (2.4) | Blessing (1972) ^68^ |
| *Stenella attenuata* | 25.4 (1.5) | Castellini & Somero (1981) ^69^ |
| *Stenella coeruleoabla* | 58 (3.4) | Blessing (1972) ^68^ |
| *Stenella longirostris* | 55 (3.2) | Dolar et al. (1999) ^70^ |
| *Lagenodelphis hosei* | 71 (4.2) | Dolar et al. (1999) ^70^ |
| *Lagenorhynchus obliquidens* | 35 (2.1) | Noren & Williams (2000) ^62^ |
| *Delphinapterus leucas* | 34 (2.0) | Noren & Williams (2000) ^62^ |
| *Lissodelphis borealis* | 18 (1.1) | Noren & Williams (2000) ^62^ |
| *Platanista indi* | 26 (1.5) | Blessing (1972) ^68^ |
| *Sousa chinensis* | 25 (1.5) | Harrison & Davis (1998) ^66^ |
| CARNIVORA |  |  |
| *Phoca vitulina* | 43.6 (2.6) | Castellini & Somero (1981) ^69^ |
| *Phoca groenlandica* | 86 (5.1) | Burns et al. (2007) ^71^ |
| *Histriophoca fasciata* | 81.0 (4.8) | Lenfant et al. (1970) ^72^ |
| *Pusa hispida* | 71.8 (4.2) | O'Brien et al. (1992) ^73^ |
| *Halichoerus grypus* | 41.0 (2.4) | Scholander (1940) ^65^ |
| *Cystophora cristata* | 95 (5.6) | Burns et al. (2007) ^71^ |
| *Phoca sibirica* | 60 (3.5) | Neshumova & Cherepanova (1984) ^74^ |
| *Mirounga angustirostris* | 65 (3.8) | Thorson & Le Boeuf (1994) ^75^ |
| *Leptonychotes weddelli* | 54 (3.2) | Ponganis et al. (1993) ^76^  Fujise et al. (1985) ^77^ |
| *Zalophus californianus* | 27-54 (1.6-3.2) | Weise & Costa (2007) ^78^  Ponganis et al. (1997) ^79^ |
| *Neophoca cinerea* | 27 (1.6) | Fowler et al. (2007) ^80^ |
| *Callorhinus ursinus* | 35.8 (2.1) | Shero et al. (2012) ^81^ |
| *Eumetopias jubata* | 27 (1.6) | Richmond & Burns (2006) ^82^ |
| *Odobenus rosmarus* | 30 (1.8) | Lenfant et al. (1970) ^72^ |
| *Enhydra lutris* | 28.7 (1.7) | Castellini & Somero (1981) ^69^ |
| SIRENIA |  |  |
| *Trichechus manatus* | 4 (0.2) | Blessing (1972) ^68^ |
| RODENTIA |  |  |
| *Castor canadensis* | 12 (0.7) | McKean & Carlton (1977) ^83^ |
| *Ondatra zibethica* | 13 (0.8) | Binkley (1980) ^84^  MacArthur (1990)^85^ |
| TERRESTRIAL SPECIES |  |  |
| Man | 2.8 (0.16) | Kragten (1996) ^86^ |
| Rat | 3.0 (0.18) | Lechner (1976) ^87^ |
| Dog | 3.3 (0.20) | Drabkin (1950) ^88^  Castellini & Somero (1981) ^69^ |
| Pig | 1.7 (0.10) | Castellini & Somero (1981) ^69^ |
| Ox (beef) | 4.5 (0.26) | Castellini & Somero (1981) ^69^ |
| Horse | 7.4 (0.44) | Drabkin (1950) ^88^ |

^a^This table was made by revision of Table 2 of Snyder (1983) ^89^ with newer data.

**Table S2. Inferred amino acid residues of ancestral Mbs^a^**

| ResNo. | swMb | aMbWp | | | | | | aMbWb’ | | | | | | aMbWb | | | | | |
| --- | --- | --- | --- | --- | --- | --- | --- | --- | --- | --- | --- | --- | --- | --- | --- | --- | --- | --- | --- |
|  |  | AA1 | PP1 | AA2 | PP2 | AA3 | PP3 | AA1 | PP1 | AA2 | PP2 | AA3 | PP3 | AA1 | PP1 | AA2 | PP2 | AA3 | PP3 |
| 0 | M | M | 1.000 |  |  |  |  | M | 1.000 |  |  |  |  | M | 1.000 |  |  |  |  |
| 1 | V | G | 1.000 |  |  |  |  | G | 1.000 |  |  |  |  | V | 1.000 |  |  |  |  |
| 2 | L | L | 1.000 |  |  |  |  | L | 1.000 |  |  |  |  | L | 1.000 |  |  |  |  |
| 3 | S | S | 1.000 |  |  |  |  | S | 1.000 |  |  |  |  | S | 1.000 |  |  |  |  |
| 4 | E | D | 1.000 |  |  |  |  | D | 1.000 |  |  |  |  | D | 1.000 |  |  |  |  |
| 5 | G | G | 1.000 |  |  |  |  | G | 1.000 |  |  |  |  | G | 1.000 |  |  |  |  |
| 6 | E | E | 1.000 |  |  |  |  | E | 1.000 |  |  |  |  | E | 1.000 |  |  |  |  |
| 7 | W | W | 1.000 |  |  |  |  | W | 1.000 |  |  |  |  | W | 1.000 |  |  |  |  |
| 8 | Q | Q | 1.000 |  |  |  |  | Q | 1.000 |  |  |  |  | Q | 1.000 |  |  |  |  |
| 9 | L | L | 1.000 |  |  |  |  | L | 1.000 |  |  |  |  | L | 1.000 |  |  |  |  |
| 10 | V | V | 1.000 |  |  |  |  | V | 1.000 |  |  |  |  | V | 1.000 |  |  |  |  |
| 11 | L | L | 1.000 |  |  |  |  | L | 1.000 |  |  |  |  | L | 1.000 |  |  |  |  |
| 12 | H | N | 1.000 |  |  |  |  | N | 1.000 |  |  |  |  | N | 1.000 |  |  |  |  |
| 13 | V | V | 0.991 | I | 0.009 |  |  | I | 0.976 | V | 0.024 |  |  | I | 1.000 |  |  |  |  |
| 14 | W | W | 1.000 |  |  |  |  | W | 1.000 |  |  |  |  | W | 1.000 |  |  |  |  |
| 15 | A | G | 0.999 | A | 0.001 |  |  | G | 0.992 | A | 0.008 |  |  | A | 1.000 |  |  |  |  |
| 16 | K | K | 1.000 |  |  |  |  | K | 1.000 |  |  |  |  | K | 1.000 |  |  |  |  |
| 17 | V | V | 1.000 |  |  |  |  | V | 1.000 |  |  |  |  | V | 1.000 |  |  |  |  |
| 18 | E | E | 1.000 |  |  |  |  | E | 1.000 |  |  |  |  | E | 1.000 |  |  |  |  |
| 19 | A | A | 1.000 |  |  |  |  | A | 1.000 |  |  |  |  | A | 1.000 |  |  |  |  |
| 20 | D | D | 1.000 |  |  |  |  | D | 1.000 |  |  |  |  | D | 1.000 |  |  |  |  |
| 21 | V | V | 1.000 |  |  |  |  | V | 0.809 | L | 0.191 |  |  | V | 1.000 |  |  |  |  |
| 22 | A | A | 1.000 |  |  |  |  | A | 1.000 |  |  |  |  | A | 1.000 |  |  |  |  |
| 23 | G | G | 1.000 |  |  |  |  | G | 1.000 |  |  |  |  | G | 1.000 |  |  |  |  |
| 24 | H | H | 1.000 |  |  |  |  | H | 1.000 |  |  |  |  | H | 1.000 |  |  |  |  |
| 25 | G | G | 1.000 |  |  |  |  | G | 1.000 |  |  |  |  | G | 1.000 |  |  |  |  |
| 26 | Q | Q | 1.000 |  |  |  |  | Q | 1.000 |  |  |  |  | Q | 1.000 |  |  |  |  |
| 27 | D | E | 0.996 | D | 0.004 |  |  | D | 0.948 | E | 0.052 |  |  | D | 1.000 |  |  |  |  |
| 28 | I | V | 1.000 |  |  |  |  | V | 0.952 | I | 0.048 |  |  | V | 1.000 |  |  |  |  |
| 29 | L | L | 1.000 |  |  |  |  | L | 1.000 |  |  |  |  | L | 1.000 |  |  |  |  |
| 30 | I | I | 1.000 |  |  |  |  | I | 1.000 |  |  |  |  | I | 1.000 |  |  |  |  |
| 31 | R | R | 1.000 |  |  |  |  | R | 1.000 |  |  |  |  | R | 1.000 |  |  |  |  |
| 32 | L | L | 1.000 |  |  |  |  | L | 1.000 |  |  |  |  | L | 1.000 |  |  |  |  |
| 33 | F | F | 1.000 |  |  |  |  | F | 1.000 |  |  |  |  | F | 1.000 |  |  |  |  |
| 34 | K | T | 0.999 | K | 0.001 |  |  | K | 0.999 |  |  |  |  | K | 1.000 |  |  |  |  |
| 35 | S | G | 1.000 |  |  |  |  | G | 1.000 |  |  |  |  | G | 1.000 |  |  |  |  |
| 36 | H | H | 1.000 |  |  |  |  | H | 1.000 |  |  |  |  | H | 1.000 |  |  |  |  |
| 37 | P | P | 1.000 |  |  |  |  | P | 1.000 |  |  |  |  | P | 1.000 |  |  |  |  |
| 38 | E | E | 1.000 |  |  |  |  | E | 1.000 |  |  |  |  | E | 1.000 |  |  |  |  |
| 39 | T | T | 1.000 |  |  |  |  | T | 1.000 |  |  |  |  | T | 1.000 |  |  |  |  |
| 40 | L | L | 1.000 |  |  |  |  | L | 1.000 |  |  |  |  | L | 1.000 |  |  |  |  |
| 41 | E | E | 1.000 |  |  |  |  | E | 1.000 |  |  |  |  | E | 1.000 |  |  |  |  |
| 42 | K | K | 1.000 |  |  |  |  | K | 1.000 |  |  |  |  | K | 1.000 |  |  |  |  |
| 43 | F | F | 1.000 |  |  |  |  | F | 1.000 |  |  |  |  | F | 1.000 |  |  |  |  |
| 44 | D | D | 0.994 | E | 0.006 |  |  | D | 1.000 |  |  |  |  | D | 1.000 |  |  |  |  |
| 45 | R | K | 1.000 |  |  |  |  | K | 1.000 |  |  |  |  | K | 1.000 |  |  |  |  |
| 46 | F | F | 1.000 |  |  |  |  | F | 1.000 |  |  |  |  | F | 1.000 |  |  |  |  |
| 47 | K | K | 1.000 |  |  |  |  | K | 1.000 |  |  |  |  | K | 1.000 |  |  |  |  |
| 48 | H | H | 0.997 | N | 0.003 |  |  | H | 1.000 |  |  |  |  | H | 1.000 |  |  |  |  |
| 49 | L | L | 1.000 |  |  |  |  | L | 1.000 |  |  |  |  | L | 1.000 |  |  |  |  |
| 50 | K | K | 1.000 |  |  |  |  | K | 1.000 |  |  |  |  | K | 1.000 |  |  |  |  |
| 51 | T | T | 1.000 |  |  |  |  | T | 1.000 |  |  |  |  | T | 1.000 |  |  |  |  |
| 52 | E | E | 1.000 |  |  |  |  | E | 1.000 |  |  |  |  | E | 1.000 |  |  |  |  |
| 53 | A | D | 0.999 | A | 0.001 |  |  | A | 0.999 | D | 0.001 |  |  | A | 1.000 |  |  |  |  |
| 54 | E | E | 1.000 |  |  |  |  | E | 1.000 |  |  |  |  | E | 1.000 |  |  |  |  |
| 55 | M | M | 1.000 |  |  |  |  | M | 1.000 |  |  |  |  | M | 1.000 |  |  |  |  |
| 56 | K | K | 0.992 | R | 0.008 |  |  | K | 1.000 |  |  |  |  | K | 1.000 |  |  |  |  |
| 57 | A | A | 1.000 |  |  |  |  | A | 1.000 |  |  |  |  | A | 1.000 |  |  |  |  |
| 58 | S | S | 0.998 | C | 0.002 |  |  | S | 1.000 |  |  |  |  | S | 1.000 |  |  |  |  |
| 59 | E | E | 1.000 |  |  |  |  | E | 1.000 |  |  |  |  | E | 1.000 |  |  |  |  |
| 60 | D | D | 0.996 | N | 0.004 |  |  | D | 1.000 |  |  |  |  | D | 1.000 |  |  |  |  |
| 61 | L | L | 1.000 |  |  |  |  | L | 1.000 |  |  |  |  | L | 1.000 |  |  |  |  |
| 62 | K | K | 1.000 |  |  |  |  | K | 1.000 |  |  |  |  | K | 1.000 |  |  |  |  |
| 63 | K | K | 1.000 |  |  |  |  | K | 1.000 |  |  |  |  | K | 1.000 |  |  |  |  |
| 64 | H | H | 1.000 |  |  |  |  | H | 1.000 |  |  |  |  | H | 1.000 |  |  |  |  |
| 65 | G | G | 1.000 |  |  |  |  | G | 1.000 |  |  |  |  | G | 1.000 |  |  |  |  |
| 66 | V | N | 1.000 |  |  |  |  | N | 1.000 |  |  |  |  | N | 1.000 |  |  |  |  |
| 67 | T | T | 1.000 |  |  |  |  | T | 1.000 |  |  |  |  | T | 1.000 |  |  |  |  |
| 68 | V | V | 1.000 |  |  |  |  | V | 1.000 |  |  |  |  | V | 1.000 |  |  |  |  |
| 69 | L | L | 1.000 |  |  |  |  | L | 1.000 |  |  |  |  | L | 1.000 |  |  |  |  |
| 70 | T | T | 1.000 |  |  |  |  | T | 1.000 |  |  |  |  | T | 1.000 |  |  |  |  |
| 71 | A | A | 1.000 |  |  |  |  | A | 1.000 |  |  |  |  | A | 1.000 |  |  |  |  |
| 72 | L | L | 1.000 |  |  |  |  | L | 1.000 |  |  |  |  | L | 1.000 |  |  |  |  |
| 73 | G | G | 1.000 |  |  |  |  | G | 1.000 |  |  |  |  | G | 1.000 |  |  |  |  |
| 74 | A | G | 1.000 |  |  |  |  | G | 1.000 |  |  |  |  | G | 1.000 |  |  |  |  |
| 75 | I | I | 1.000 |  |  |  |  | I | 1.000 |  |  |  |  | I | 1.000 |  |  |  |  |
| 76 | L | L | 1.000 |  |  |  |  | L | 1.000 |  |  |  |  | L | 1.000 |  |  |  |  |
| 77 | K | K | 1.000 |  |  |  |  | K | 1.000 |  |  |  |  | K | 1.000 |  |  |  |  |
| 78 | K | K | 1.000 |  |  |  |  | K | 1.000 |  |  |  |  | K | 1.000 |  |  |  |  |
| 79 | K | K | 1.000 |  |  |  |  | K | 1.000 |  |  |  |  | K | 1.000 |  |  |  |  |
| 80 | G | G | 1.000 |  |  |  |  | G | 1.000 |  |  |  |  | G | 1.000 |  |  |  |  |
| 81 | H | H | 1.000 |  |  |  |  | H | 1.000 |  |  |  |  | H | 1.000 |  |  |  |  |
| 82 | H | H | 1.000 |  |  |  |  | H | 1.000 |  |  |  |  | H | 1.000 |  |  |  |  |
| 83 | E | E | 1.000 |  |  |  |  | E | 0.994 | D | 0.006 |  |  | E | 1.000 |  |  |  |  |
| 84 | A | A | 0.998 | E | 0.002 |  |  | A | 1.000 |  |  |  |  | A | 1.000 |  |  |  |  |
| 85 | E | E | 1.000 |  |  |  |  | E | 1.000 |  |  |  |  | E | 1.000 |  |  |  |  |
| 86 | L | L | 1.000 |  |  |  |  | L | 1.000 |  |  |  |  | L | 1.000 |  |  |  |  |
| 87 | K | K | 1.000 |  |  |  |  | K | 1.000 |  |  |  |  | K | 1.000 |  |  |  |  |
| 88 | P | P | 1.000 |  |  |  |  | P | 1.000 |  |  |  |  | P | 1.000 |  |  |  |  |
| 89 | L | L | 1.000 |  |  |  |  | L | 1.000 |  |  |  |  | L | 1.000 |  |  |  |  |
| 90 | A | A | 1.000 |  |  |  |  | A | 1.000 |  |  |  |  | A | 1.000 |  |  |  |  |
| 91 | Q | Q | 0.997 | H | 0.003 |  |  | Q | 1.000 |  |  |  |  | Q | 1.000 |  |  |  |  |
| 92 | S | S | 1.000 |  |  |  |  | S | 1.000 |  |  |  |  | S | 1.000 |  |  |  |  |
| 93 | H | H | 1.000 |  |  |  |  | H | 1.000 |  |  |  |  | H | 1.000 |  |  |  |  |
| 94 | A | A | 1.000 |  |  |  |  | A | 1.000 |  |  |  |  | A | 1.000 |  |  |  |  |
| 95 | T | T | 1.000 |  |  |  |  | T | 1.000 |  |  |  |  | T | 1.000 |  |  |  |  |
| 96 | K | K | 1.000 |  |  |  |  | K | 1.000 |  |  |  |  | K | 1.000 |  |  |  |  |
| 97 | H | H | 1.000 |  |  |  |  | H | 1.000 |  |  |  |  | H | 1.000 |  |  |  |  |
| 98 | K | K | 1.000 |  |  |  |  | K | 1.000 |  |  |  |  | K | 1.000 |  |  |  |  |
| 99 | I | I | 1.000 |  |  |  |  | I | 1.000 |  |  |  |  | I | 1.000 |  |  |  |  |
| 100 | P | P | 1.000 |  |  |  |  | P | 1.000 |  |  |  |  | P | 1.000 |  |  |  |  |
| 101 | I | I | 0.925 | V | 0.075 |  |  | I | 1.000 |  |  |  |  | I | 1.000 |  |  |  |  |
| 102 | K | K | 1.000 |  |  |  |  | K | 1.000 |  |  |  |  | K | 1.000 |  |  |  |  |
| 103 | Y | Y | 1.000 |  |  |  |  | Y | 1.000 |  |  |  |  | Y | 1.000 |  |  |  |  |
| 104 | L | L | 1.000 |  |  |  |  | L | 1.000 |  |  |  |  | L | 1.000 |  |  |  |  |
| 105 | E | E | 1.000 |  |  |  |  | E | 1.000 |  |  |  |  | E | 1.000 |  |  |  |  |
| 106 | F | F | 1.000 |  |  |  |  | F | 1.000 |  |  |  |  | F | 1.000 |  |  |  |  |
| 107 | I | I | 1.000 |  |  |  |  | I | 1.000 |  |  |  |  | I | 1.000 |  |  |  |  |
| 108 | S | S | 1.000 |  |  |  |  | S | 1.000 |  |  |  |  | S | 1.000 |  |  |  |  |
| 109 | E | D | 0.988 | E | 0.012 |  |  | D | 0.652 | E | 0.348 |  |  | D | 1.000 |  |  |  |  |
| 110 | A | A | 1.000 |  |  |  |  | A | 1.000 |  |  |  |  | A | 1.000 |  |  |  |  |
| 111 | I | I | 1.000 |  |  |  |  | I | 1.000 |  |  |  |  | I | 1.000 |  |  |  |  |
| 112 | I | I | 1.000 |  |  |  |  | I | 1.000 |  |  |  |  | I | 1.000 |  |  |  |  |
| 113 | H | H | 1.000 |  |  |  |  | H | 1.000 |  |  |  |  | H | 1.000 |  |  |  |  |
| 114 | V | V | 1.000 |  |  |  |  | V | 1.000 |  |  |  |  | V | 1.000 |  |  |  |  |
| 115 | L | L | 1.000 |  |  |  |  | L | 1.000 |  |  |  |  | L | 1.000 |  |  |  |  |
| 116 | H | Q | 0.998 | H | 0.002 |  |  | H | 0.998 | Q | 0.002 |  |  | H | 1.000 |  |  |  |  |
| 117 | S | S | 1.000 |  |  |  |  | S | 1.000 |  |  |  |  | S | 1.000 |  |  |  |  |
| 118 | R | K | 0.994 | R | 0.006 |  |  | R | 0.999 | K | 0.001 |  |  | R | 1.000 |  |  |  |  |
| 119 | H | H | 1.000 |  |  |  |  | H | 1.000 |  |  |  |  | H | 1.000 |  |  |  |  |
| 120 | P | P | 1.000 |  |  |  |  | P | 1.000 |  |  |  |  | P | 1.000 |  |  |  |  |
| 121 | G | G | 1.000 |  |  |  |  | G | 1.000 |  |  |  |  | G | 1.000 |  |  |  |  |
| 123 | D | D | 1.000 |  |  |  |  | D | 1.000 |  |  |  |  | D | 1.000 |  |  |  |  |
| 124 | F | F | 1.000 |  |  |  |  | F | 1.000 |  |  |  |  | F | 1.000 |  |  |  |  |
| 125 | G | G | 1.000 |  |  |  |  | G | 1.000 |  |  |  |  | G | 1.000 |  |  |  |  |
| 126 | A | A | 1.000 |  |  |  |  | A | 1.000 |  |  |  |  | A | 1.000 |  |  |  |  |
| 127 | D | D | 1.000 |  |  |  |  | D | 1.000 |  |  |  |  | D | 1.000 |  |  |  |  |
| 129 | A | A | 1.000 |  |  |  |  | A | 1.000 |  |  |  |  | A | 1.000 |  |  |  |  |
| 130 | Q | Q | 1.000 |  |  |  |  | Q | 1.000 |  |  |  |  | Q | 1.000 |  |  |  |  |
| 131 | G | G | 1.000 |  |  |  |  | G | 1.000 |  |  |  |  | G | 1.000 |  |  |  |  |
| 132 | A | A | 1.000 |  |  |  |  | A | 1.000 |  |  |  |  | A | 1.000 |  |  |  |  |
| 133 | M | M | 1.000 |  |  |  |  | M | 1.000 |  |  |  |  | M | 1.000 |  |  |  |  |
| 134 | N | N | 1.000 |  |  |  |  | N | 1.000 |  |  |  |  | N | 1.000 |  |  |  |  |
| 135 | K | K | 1.000 |  |  |  |  | K | 1.000 |  |  |  |  | K | 1.000 |  |  |  |  |
| 136 | A | A | 1.000 |  |  |  |  | A | 1.000 |  |  |  |  | A | 1.000 |  |  |  |  |
| 137 | L | L | 1.000 |  |  |  |  | L | 1.000 |  |  |  |  | L | 1.000 |  |  |  |  |
| 138 | E | E | 1.000 |  |  |  |  | E | 1.000 |  |  |  |  | E | 1.000 |  |  |  |  |
| 139 | L | L | 1.000 |  |  |  |  | L | 1.000 |  |  |  |  | L | 1.000 |  |  |  |  |
| 140 | F | F | 1.000 |  |  |  |  | F | 1.000 |  |  |  |  | F | 1.000 |  |  |  |  |
| 141 | R | R | 1.000 |  |  |  |  | R | 1.000 |  |  |  |  | R | 1.000 |  |  |  |  |
| 142 | K | N | 0.999 | K | 0.001 |  |  | K | 0.999 | N | 0.001 |  |  | K | 1.000 |  |  |  |  |
| 143 | D | D | 1.000 |  |  |  |  | D | 1.000 |  |  |  |  | D | 1.000 |  |  |  |  |
| 144 | I | I | 0.959 | M | 0.040 | V | 0.001 | I | 1.000 |  |  |  |  | I | 1.000 |  |  |  |  |
| 145 | A | A | 1.000 |  |  |  |  | A | 1.000 |  |  |  |  | A | 1.000 |  |  |  |  |
| 146 | A | A | 1.000 |  |  |  |  | A | 1.000 |  |  |  |  | A | 1.000 |  |  |  |  |
| 147 | K | K | 1.000 |  |  |  |  | K | 1.000 |  |  |  |  | K | 1.000 |  |  |  |  |
| 148 | Y | Y | 1.000 |  |  |  |  | Y | 1.000 |  |  |  |  | Y | 1.000 |  |  |  |  |
| 149 | K | K | 1.000 |  |  |  |  | K | 1.000 |  |  |  |  | K | 1.000 |  |  |  |  |
| 150 | E | E | 1.000 |  |  |  |  | E | 1.000 |  |  |  |  | E | 1.000 |  |  |  |  |
| 151 | L | L | 1.000 |  |  |  |  | L | 1.000 |  |  |  |  | L | 1.000 |  |  |  |  |
| 152 | G | G | 1.000 |  |  |  |  | G | 1.000 |  |  |  |  | G | 1.000 |  |  |  |  |
| 153 | Y | F | 1.000 |  |  |  |  | F | 1.000 |  |  |  |  | F | 1.000 |  |  |  |  |
| 154 | Q | Q | 1.000 |  |  |  |  | Q | 0.853 | H | 0.147 |  |  | Q | 1.000 |  |  |  |  |
| 155 | G | G | 1.000 |  |  |  |  | G | 1.000 |  |  |  |  | G | 1.000 |  |  |  |  |

^a^ AA1, AA2, and AA3 are amino acids with the best, second, and third probabilities.

**Table S3.**  **Molecular properties of ancient and sperm whale Mbs based on their sequences and X-ray crystallographic structures**

| Mb | *Mr*^a^ | Formal net charges^b^ | Formal p*I*^c^ | *Z*_Mb_ ^d^  (pH 7.0) | p*I* ^d^ | Δ*G*_solv_ ^e^  kcal/mol | ΔΔ*G*_mut_ ^f^  kcal/mol |
| --- | --- | --- | --- | --- | --- | --- | --- |
| aMbWp | 17116.4 | +9 | 6.97 | +0.09 | 7.02 | -1807.6±68.0 | - |
| aMbWb’ | 17150.5 | +13 | 7.38 | +3.01 | 8.03 | -1653.9±28.5 | +0.06 |
| aMbWb | 17206.6 | +13 | 7.37 | +3.64 | 8.20 | -1633.7±48.9 | -2.56 |
| swMb | 17330.8 | +14 | 9.35 | +3.89 | 8.16 | -1623.1±51.3 | -4.41 |

^a^ Relative molecular masses of the apo proteins, which were calculated from the amino acid sequences.

^b^ Formal net charges were calculated based on the amino acid sequences assuming that the charges of the positively charged residues, Lys, Arg, His, and the negatively charged residues, Asp, Glu, are +1 and -1, respectively.

^c^ Formal isoelectric points were calculated based on the amino acid sequences, according to Skoog & Wichman (1986) ^90^.

^d^ Total net charges (*Z*_Mb_) and isoelectric points (p*I*) were calculated based on the X-ray crystallographic structures, according to Spassov & Yan (2008) ^91^.

^e^ The solvation free energies were calculated based on the X-ray structures and the MD simulations, according to Sumi et al. (2015) ^41^.

^f^ The mutational energy changes (*ΔΔG*_mut_) were calculated based on the X-ray structures, according to Spassov & Yan (2013) ^92^. The values in each line are *ΔΔG*_mut_ for the residue replacements from the proximate ancient Mb, *i.e.*, the value of aMbWb is for the replacements of G1V and G15A on aMbWb’. The *ΔΔG*_mut_ for each residue replacement and the itemized energy terms are shown in Fig. S5.

Table S4. X-ray data collection and refinement statistics^a^

| Protein name^b^ | aMbWp  (5YCG) | aMbWb’  (5YCI) | aMbWb’-IMD^c^  (5YCJ) | aMbWb  (5YCH) | swMb  (5YCE) |
| --- | --- | --- | --- | --- | --- |
| Space group | *P2_1_* | *P2_1_2_1_2_1_* | *P2_1_2_1_2_1_* | *P2_1_* | *P2_1_* |
| Cell constants  [a, b, c **Å**, (β^o^)] | 55.4, 29.7, 58.4, 112.7 | 39.3, 69.3, 108.1 | 39.2, 69.1, 108.4 | 34.4, 30.4, 63.3, 105.8 | 34.2, 30.7, 63.6, 105.5 |
| Wavelength (**Å**) | 1.0000 | 1.0000 | 0.9800 | 1.0000 | 0.8000 |
| Resolution range - data collection (**Å**) | 25.00 - 2.40  (2.53 - 2.40) | 25.00 - 1.97  (2.07 - 1.97) | 25.00 - 1.58  (1.67 - 1.58) | 25.00 - 1.35  (1.43 - 1.35) | 10.00 - 0.77  (0.81 - 0.77) |
| R_sym_ | 0.066 (0.088) | 0.058 (0.087) | 0.071 (0.112) | 0.047 (0.194) | 0.023 (0.469) |
| Completeness (%) | 99.9 (100.0) | 99.9 (99.8) | 99.9 (100.0) | 97.3 (95.4) | 99.3 (98.2) |
| <*I*/*σI*> | 22.9 (21.3) | 38.8 (33.6) | 29.4 (25.3) | 22.3. (8.9) | 20.5 (2.3) |
| Resolution range - refinement (**Å**) | 23.63 - 2.40  (3.02 - 2.40) | 25.00 - 1.97  (2.06 - 1.97) | 25.00 - 1.58  (1.62 - 1.58) | 22.40 - 1.35  (1.40 - 1.35) | 10.00 - 0.77  (0.779 - 0.770) |
| No. reflections | 7054 (3317) | 21421 (2615) | 40921 (2657) | 26832 (2584) | 148914 (4610) |
| R_cryst_ | 0.184 (0.193) | 0.171 (0.192) | 0.152 (0.149) | 0.171 (0.210) | 0.145 (0.428) |
| R_free_ | 0.227 (0.231) | 0.217 (0.238) | 0.183 (0.187) | 0.207 (0.258) | 0.162 (0.422) |
| RMSD length (**Å**) | 0.015 | 0.013 | 0.014 | 0.012 | 0.013 |
| RMSD angle (^o^) | 1.55 | 1.29 | 1.45 | 1.28 | 1.59 |

^a^ Values for the highest resolution bin are in parentheses.

^b^ In parentheses are the PDB accession codes.

^c^ Imidazole-ligated aMbWb’ structure.

**Table S5**. **Parameters obtained for Mb solubility curves with PEG-6000^a^**

| Mb | Slope (*β*) | Intercept (log *S_0_*) | *R*^2^ |
| --- | --- | --- | --- |
| aMbWp | -0.109±0.006 | 2.83±0.15 | 0.984 |
| aMbWb’ | -0.045±0.002 | 2.18±0.05 | 0.992 |
| aMbWb | -0.036±0.003 | 2.19±0.09 | 0.959 |
| swMb | -0.035±0.002 | 2.19±0.05 | 0.984 |
| hsMb | -0.088±0.004 | 2.78±0.10 | 0.991 |
| bvMb | -0.090±0.003 | 2.26±0.07 | 0.991 |

**^a^** The data were obtained from Fig. 4a.

**Table S6.**  **Thermodynamic parameters for folding reactions of ancient and sperm whale apoMbs^a^**

| Mb | Δ*G*_1_ | *m*_1_ | Δ*G*_2_ | *m*_2_ | Δ*G*_fold_ |
| --- | --- | --- | --- | --- | --- |
| aMbWp | -1.94±0.19 | 2.03±0.34 | -0.75±0.20 | 0.51±0.08 | -2.69±0.39 |
| aMbWb’ | -2.27±0.25 | 2.49±0.33 | -0.88±0.18 | 0.57±0.07 | -3.15±0.43 |
| aMbWb | -1.86±0.15 | 2.23±0.25 | -1.16±0.16 | 0.75±0.07 | -3.02±0.31 |
| swMb | -5.00±0.66 | 4.97±0.69 | -1.34±0.14 | 0.76±0.06 | -6.33±0.80 |
| hsMb | -3.55±0.25 | 3.57±0.34 | -0.10±0.20 | 0.53±0.09 | -3.65±0.44 |
| bvMb | -2.22±0.27 | 2.50±0.40 | -0.79±0.18 | 0.53±0.07 | -3.01±0.45 |

^a^ The Δ*G* and *m* values are expressed in units, kcal mol^-1^ and kcal mol^-1^ M^-1^, respectively. These parameters were obtained by the denaturation experiments with Gd-HCl (Fig. 4d) and the analyses assuming the three-state folding transition:

_1 2_

**N ⇄ I ⇄ U**, as described in the Methods section.

**Supplementary References**

1 Kaminuma, E. *et al.* DDBJ launches a new archive database with analytical tools for next-generation sequence data. *Nucleic Acids Res* **38**, D33-38, doi:10.1093/nar/gkp847 (2010).

2 Altschul, S. F., Gish, W., Miller, W., Myers, E. W. & Lipman, D. J. Basic local alignment search tool. *J Mol Biol* **215**, 403-410, doi:10.1016/S0022-2836(05)80360-2 (1990).

3 O'Leary, N. A. *et al.* Reference sequence (RefSeq) database at NCBI: current status, taxonomic expansion, and functional annotation. *Nucleic Acids Res* **44**, D733-745, doi:10.1093/nar/gkv1189 (2016).

4 Thompson, J. D., Gibson, T. J. & Higgins, D. G. Multiple sequence alignment using ClustalW and ClustalX. *Curr Protoc Bioinformatics* **Chapter 2**, Unit 2 3, doi:10.1002/0471250953.bi0203s00 (2002).

5 Katoh, K., Misawa, K., Kuma, K. & Miyata, T. MAFFT: a novel method for rapid multiple sequence alignment based on fast Fourier transform. *Nucleic Acids Res* **30**, 3059-3066, doi:10.1093/nar/gkf436 (2002).

6 Saitou, N. & Nei, M. The neighbor-joining method: a new method for reconstructing phylogenetic trees. *Mol Biol Evol* **4**, 406-425, doi:10.1093/oxfordjournals.molbev.a040454 (1987).

7 Jones, D. T., Taylor, W. R. & Thornton, J. M. The rapid generation of mutation data matrices from protein sequences. *Comput Appl Biosci* **8**, 275-282, doi:10.1093/bioinformatics/8.3.275 (1992).

8 Meredith, R. W. *et al.* Impacts of the cretaceous terrestrial revolution and KPg extinction on mammal diversification. *Science* **334**, 521-524, doi:10.1126/science.1211028 (2011).

9 Spaulding, M., O'Leary, M. A. & Gatesy, J. Relationships of Cetacea (Artiodactyla) among mammals: increased taxon sampling alters interpretations of key fossils and character evolution. *PLoS One* **4**, e7062, doi:10.1371/journal.pone.0007062 (2009).

10 Mirceta, S. *et al.* Evolution of mammalian diving capacity traced by myoglobin net surface charge. *Science* **340**, 1234192, doi:10.1126/science.1234192 (2013).

11 Yang, Z. PAML 4: phylogenetic analysis by maximum likelihood. *Mol Biol Evol* **24**, 1586-1591, doi:10.1093/molbev/msm088 (2007).

12 Yang, Z. & Nielsen, R. Synonymous and nonsynonymous rate variation in nuclear genes of mammals. *J Mol Evol* **46**, 409-418, doi:10.1007/PL00006320 (1998).

13 Yang, Z. & Rannala, B. Bayesian estimation of species divergence times under a molecular clock using multiple fossil calibrations with soft bounds. *Mol Biol Evol* **23**, 212-226, doi:10.1093/molbev/msj024 (2006).

14 Milinkovitch, M. C., Orti, G. & Meyer, A. Revised phylogeny of whales suggested by mitochondrial ribosomal DNA sequences. *Nature* **361**, 346-348, doi:10.1038/361346a0 (1993).

15 Milinkovitch, M. C., Orti, G. & Meyer, A. Novel phylogeny of whales revisited but not revised. *Mol Biol Evol* **12**, 518-520, doi:10.1093/oxfordjournals.molbev.a040226 (1995).

16 Cassens, I. *et al.* Independent adaptation to riverine habitats allowed survival of ancient cetacean lineages. *Proc Natl Acad Sci U S A* **97**, 11343-11347, doi:10.1073/pnas.97.21.11343 (2000).

17 Marx, F. G. & Fordyce, R. E. Baleen boom and bust: a synthesis of mysticete phylogeny, diversity and disparity. *R Soc Open Sci* **2**, 140434, doi:10.1098/rsos.140434 (2015).

18 Nikaido, M. *et al.* Retroposon analysis of major cetacean lineages: the monophyly of toothed whales and the paraphyly of river dolphins. *Proc Natl Acad Sci U S A* **98**, 7384-7389, doi:10.1073/pnas.121139198 (2001).

19 Nikaido, M., Piskurek, O. & Okada, N. Toothed whale monophyly reassessed by SINE insertion analysis: the absence of lineage sorting effects suggests a small population of a common ancestral species. *Mol Phylogenet Evol* **43**, 216-224, doi:10.1016/j.ympev.2006.08.005 (2007).

20 Gingerich, P. D., Smith, B. H. & Simons, E. L. Hind limbs of Eocene basilosaurus: evidence of feet in whales. *Science* **249**, 154-157, doi:10.1126/science.249.4965.154 (1990).

21 Thewissen, J. G., Hussain, S. T. & Arif, M. Fossil evidence for the origin of aquatic locomotion in archaeocete whales. *Science* **263**, 210-212, doi:10.1126/science.263.5144.210 (1994).

22 Bajpai, S. & Gingerich, P. D. A new Eocene archaeocete (Mammalia, Cetacea) from India and the time of origin of whales. *Proc Natl Acad Sci U S A* **95**, 15464-15468, doi:10.1073/pnas.95.26.15464 (1998).

23 Antonini, E. & Brunori, M. *Hemoglobin and myoglobin in their reactions with ligands*. (American Elsevier, 1971).

24 Wittenberg, J. B. & Wittenberg, B. A. in *Methods in Enzymology* Vol. 76 (eds Eraldo Antonini, Luigi Rossi-Bernardi, & Emilia Chiancone) 29-42 (Academic Press, 1981).

25 Hayashi, A., Suzuki, T. & Shin, M. An enzymic reduction system for metmyoglobin and methemoglobin, and its application to functional studies of oxygen carriers. *Biochim Biophys Acta* **310**, 309-316, doi:10.1016/0005-2795(73)90110-4 (1973).

26 Bowen, W. J. The absorption spectra and extinction coefficients of myoglobin. *J Biol Chem* **179**, 235-245 (1949).

27 Watson, H. C. The stereochemistry of the protein myoglobin. *Prog Stereochem* **4**, 299 (1969).

28 Otwinowski, Z. & Minor, W. in *Methods in Enzymology, Volume 276: Macromolecular Crystallography, part A* Vol. 276 (eds C.W. Carter Jr. & R. M. Sweet) 307-326 (1997).

29 Battye, T. G., Kontogiannis, L., Johnson, O., Powell, H. R. & Leslie, A. G. iMOSFLM: a new graphical interface for diffraction-image processing with MOSFLM. *Acta Crystallogr D Biol Crystallogr* **67**, 271-281, doi:10.1107/S0907444910048675 (2011).

30 Adams, P. D. *et al.* PHENIX: a comprehensive Python-based system for macromolecular structure solution. *Acta Crystallogr D Biol Crystallogr* **66**, 213-221, doi:10.1107/S0907444909052925 (2010).

31 Vagin, A. & Teplyakov, A. Molecular replacement with MOLREP. *Acta Crystallogr D Biol Crystallogr* **66**, 22-25, doi:10.1107/S0907444909052925 (2010).

32 Emsley, P., Lohkamp, B., Scott, W. G. & Cowtan, K. Features and development of Coot. *Acta Crystallogr D Biol Crystallogr* **66**, 486-501, doi:10.1107/S0907444910007493 (2004).

33 Laskowski, R. A., Moss, D. S. & Thornton, J. M. Main-chain bond lengths and bond angles in protein structures. *J Mol Biol* **231**, 1049-1067, doi:10.1006/jmbi.1993.1351 (1993).

34 Pettersen, E. F. *et al.* UCSF Chimera--a visualization system for exploratory research and analysis. *J Comput Chem* **25**, 1605-1612, doi:10.1002/jcc.20084 (2004).

35 Hornak, V. *et al.* Comparison of multiple Amber force fields and development of improved protein backbone parameters. *Proteins* **65**, 712-725, doi:10.1002/prot.21123 (2006).

36 Case, D. A. *et al.* *AMBER 12*. (University of California, 2012).

37 Arcon, J. P., Rosi, P., Petruk, A. A., Marti, M. A. & Estrin, D. A. Molecular mechanism of myoglobin autoxidation: insights from computer simulations. *J Phys Chem B* **119**, 1802-1813, doi:10.1021/jp5093948 (2015).

38 McLellan, T. Molecular charge and electrophoretic mobility in cetacean myoglobins of known sequence. *Biochem Genet* **22**, 181-200, doi:10.1007/BF00499297 (1984).

39 Darden, T., York, D. & Pedersen, L. Particle mesh Ewald: An N·log(N) method for Ewald sums in large systems. *J Chem Phys* **98**, 10089-10092, doi:doi:http://dx.doi.org/10.1063/1.464397 (1993).

40 Berendsen, H. J. C., Postma, J. P. M., van Gunsteren, W. F., DiNola, A. & Haak, J. R. Molecular dynamics with coupling to an external bath. *J Chem Phys* **81**, 3684-3690, doi:doi:http://dx.doi.org/10.1063/1.448118 (1984).

41 Sumi, T., Mitsutake, A. & Maruyama, Y. A solvation-free-energy functional: a reference-modified density functional formulation. *J Comput Chem* **36**, 1359-1369, doi:10.1002/jcc.23942 (2015).

42 Sumi, T., Maruyama, Y., Mitsutake, A. & Koga, K. A reference-modified density functional theory: An application to solvation free-energy calculations for a Lennard-Jones solution. *J Chem Phys* **144**, 224104, doi:10.1063/1.4953191 (2016).

43 Sumi, T., Maruyama, Y., Mitsutake, A., Mochizuki, K. & Koga, K. Application of reference-modified density functional theory: Temperature and pressure dependences of solvation free energy. *J Comput Chem* **39**, 202-217, doi:10.1002/jcc.25101 (2018).

44 Kovalenko, A. & Hirata, F. Self-consistent description of a metal-water interface by the Kohn-Sham density functional theory and the three-dimensional reference interaction site model. *J Chem Phys* **110**, 10095-10112, doi:10.1063/1.478883 (1999).

45 Jorgensen, W. L., Chandrasekhar, J., Madura, J. D., Impey, R. W. & Klein, M. L. Comparison of simple potential functions for simulating liquid water. *J Chem Phys* **79**, 926-935, doi:10.1063/1.445869 (1983).

46 Pettitt, B. M. & Rossky, P. J. Integral equation predictions of liquid state structure for waterlike intermolecular potentials. *J Chem Phys* **77**, 1451-1457, doi:10.1063/1.443972 (1982).

47 Sumi, T. & Sekino, H. A Self-Consistent Density-Functional Approach for Homogeneous and Inhomogeneous Classical Fluids. *Journal of the Physical Society of Japan* **77**, 034605-034605, doi:10.1143/jpsj.77.034605 (2008).

48 Maruyama, Y. & Hirata, F. Modified anderson method for accelerating 3D-RISM calculations using graphics processing unit. *J Chem Theory Comput* **8**, 3015-3021, doi:10.1021/ct300355r (2012).

49 Walenta, E. Small angle X-ray scattering. *Acta Polymerica* **36**, 296-296, doi:10.1002/actp.1985.010360520 (1985).

50 Kramer, R. M., Shende, V. R., Motl, N., Pace, C. N. & Scholtz, J. M. Toward a molecular understanding of protein solubility: increased negative surface charge correlates with increased solubility. *Biophys J* **102**, 1907-1915, doi:10.1016/j.bpj.2012.01.060 (2012).

51 Ilavsky, J. Nika: software for two-dimensional data reduction. *J Appl Crystallogr* **45**, 324-328, doi:10.1107/S0021889812004037 (2012).

52 Mylonas, E. & Svergun, D. I. Accuracy of molecular mass determination of proteins in solution by small-angle X-ray scattering. *J Appl Crystallogr* **40**, s245-s249, doi:10.1107/S002188980700252X (2007).

53 Orthaber, D., Bergmann, A. & Glatter, O. SAXS experiments on absolute scale with Kratky systems using water as a secondary standard. *J Appl Crystallogr* **33**, 218-225, doi:doi:10.1107/S0021889899015216 (2000).

54 Morimoto, M. *et al.* Asphaltene aggregation behavior in bromobenzene determined by small-angle X-ray scattering. *Energy & Fuels* **29**, 5737-5743, doi:10.1021/acs.energyfuels.5b01491 (2015).

55 Glatter, O. & Kratky, O. *Small angle X-ray scattering*. (Academic Press, 1982).

56 Zimm, B. H. The scattering of light and the radial distribution function of high polymer solutions. *J Chem Phys* **16**, 1093-1099, doi:10.1063/1.1746738 (1948).

57 Goldenberg, D. P. & Argyle, B. Self crowding of globular proteins studied by small-angle x-ray scattering. *Biophys J* **106**, 895-904, doi:10.1016/j.bpj.2013.12.004 (2014).

58 Bonneté, F., Finet, S. & Tardieu, A. Second virial coefficient: variations with lysozyme crystallization conditions. *J Cryst Growth* **196**, 403-414, doi:10.1016/S0022-0248(98)00826-4 (1999).

59 Barrick, D. & Baldwin, R. L. Three-state analysis of sperm whale apomyoglobin folding. *Biochemistry* **32**, 3790-3796, doi:10.1021/bi00065a035 (1993).

60 Isogai, Y., Ishii, A., Fujisawa, T., Ota, M. & Nishikawa, K. Redesign of artificial globins: effects of residue replacements at hydrophobic sites on the structural properties. *Biochemistry* **39**, 5683-5690, doi:10.1021/bi992687+ (2000).

61 Isogai, Y. Native protein sequences are designed to destabilize folding intermediates. *Biochemistry* **45**, 2488-2492, doi:10.1021/bi0523714 (2006).

62 Noren, S. R. & Williams, T. M. Body size and skeletal muscle myoglobin of cetaceans: adaptations for maximizing dive duration. *Comp Biochem Physiol A Mol Integr Physiol* **126**, 181-191, doi:10.1016/S1095-6433(00)00182-3 (2000).

63 Tawara, T. On the respiratory pigments of whale (Studies on Whale Blood II). *Scientific Rep Whales Res Inst* **3**, 96-101 (1950).

64 Lockyer, C. Body weights of some species of large whales. *ICES Journal of Marine Science* **36**, 259-273, doi:10.1093/icesjms/36.3.259 (1976).

65 Scholander, P. F. Experimental investigations on the respiratory function in diving mammals and birds. *Hvalradets Skr* **22**, 1-131 (1940).

66 Harrison, L. K. & Davis, R. W. in *World Marine Mammal Science Conference* 60 (Society of Marine Mammalogy, Monaco, 1998).

67 Blessing, M. H. & Hartschen-Niemeyer, E. Über den Myoglobingehalt der Herz und Skelettmuskulatur insbesondere einiger mariner Sauger. *Z Biol* **116**, 302-313 (1969).

68 Blessing, M. H. Myoglobin concentration in Platanista indi. *Invest Cetacea* **4**, 91-92 (1972).

69 Castellini, M. A. & Somero, G. N. Buffering capacity of vertebrate muscle: Correlations with potentials for anaerobic function. *J Comp Physiol* **143**, 191-198, doi:10.1007/BF00797698 (1981).

70 Dolar, M. L., Suarez, P., Ponganis, P. J. & Kooyman, G. L. Myoglobin in pelagic small cetaceans. *J Exp Biol* **202**, 227-236 (1999).

71 Burns, J. M., Lestyk, K. C., Folkow, L. P., Hammill, M. O. & Blix, A. S. Size and distribution of oxygen stores in harp and hooded seals from birth to maturity. *J Comp Physiol B* **177**, 687-700, doi:10.1007/s00360-007-0167-2 (2007).

72 Lenfant, C., Johansen, K. & Torrance, J. D. Gas transport and oxygen storage capacity in some pinnipeds and the sea otter. *Respir Physiol* **9**, 277-286, doi:10.1016/0034-5687(70)90076-9 (1970).

73 O'Brien, P. J. *et al.* Rapid, simple and sensitive microassay for skeletal and cardiac muscle myoglobin and hemoglobin: use in various animals indicates functional role of myohemoproteins. *Mol Cell Biochem* **112**, 45-52, doi:10.1007/BF00229642 (1992).

74 Neshumova, T. V., Cherepanova, V. A. & Petrov, E. A. Myoglobin distribution in muscles of the seal *Pusa sibirca*. . *Zh Evol Biokhim Fiziol* **19**, 93 (1983).

75 Thorson, P. H. & Le Boeuf, B. J. in *Population Ecology, Behavior, and Physiology* (eds B. J. Le Boeuf & R. M. Laws) 271 -289 (University of California Press, 1994).

76 Ponganis, P. J., Kooyman, G. L. & Castellini, M. A. Determinants of the aerobic dive limit of weddell seals: analysis of diving metabolic rates, postdive end tidal Po2's, and blood and muscle oxygen stores. *Physiol Zool* **66**, 732-749, doi:10.1086/physzool.66.5.30163821 (1993).

77 Fujise, Y., Hidaka, H., Tathukawa, R. & Miyazaki, N. External measurements and organ weights of five Weddell seals (*Leptonychotes weddelli*) caught near Syowa Station. *Nankyoku-Shiryou* **85**, 96-101 (1985).

78 Weise, M. J. & Costa, D. P. Total body oxygen stores and physiological diving capacity of California sea lions as a function of sex and age. *J Exp Biol* **210**, 278, doi:10.1242/jeb.02643 (2007).

79 Ponganis, P. J., Kooyman, G. L., Winter, L. M. & Starke, L. N. Heart rate and plasma lactate responses during submerged swimming and trained diving in California sea lions, Zalophus californianus. *J Comp Physiol B* **167**, 9-16, doi:10.1007/s003600050042 (1997).

80 Fowler, S. L., Costa, D. P., Arnould, J. P. Y., Gales, N. J. & Burns, J. M. Ontogeny of oxygen stores and physiological diving capability in Australian sea lions. *Funct Ecol* **21**, 922-935, doi:10.1111/j.1365-2435.2007.01295.x (2007).

81 Shero, M. R., Andrews, R. D., Lestyk, K. C. & Burns, J. M. Development of the aerobic dive limit and muscular efficiency in northern fur seals (Callorhinus ursinus). *J Comp Physiol B* **182**, 425-436, doi:10.1007/s00360-011-0619-6 (2012).

82 Richmond, J. P., Burns, J. M. & Rea, L. D. Ontogeny of total body oxygen stores and aerobic dive potential in Steller sea lions (*Eumetopias jubatus*). *J Comp Physiol B* **176**, 535-545, doi:10.1007/s00360-006-0076-9 (2006).

83 McKean, T. & Carlton, C. Oxygen storage in beavers. *J Appl Physiol Respir Environ Exerc Physiol* **42**, 545-547, doi:10.1152/jappl.1977.42.4.545 (1977).

84 Binkley, E. L. *Adaptations to diving in the muskrat.* M.S. thesis, University of Colorado, (1980).

85 MacArthur, R. A. Seasonal changes in the oxygen storage capacity and aerobic dive limits of the muskrat (Ondatra zibethicus). *Journal of Comparative Physiology B* **160**, 593-599, doi:10.1007/BF00258987 (1990).

86 Kragten, J. A. *et al.* Distribution of myoglobin and fatty acid-binding protein in human cardiac autopsies. *Clin Chem* **42**, 337-338 (1996).

87 Lechner, A. J. Respiratory adaptations in burrowing pocket gophers from sea level and high altitude. *J Appl Physiol* **41**, 168-173, doi:10.1152/jappl.1976.41.2.168 (1976).

88 Drabkin, D. L. The distribution of the chromoproteins, hemoglobin, myoglobin, and cytochrome C, in the tissues of different species, and the relationship of the total content of each chromoprotein to body mass. *J Biol Chem* **182**, 317-334 (1950).

89 Snyder, G. K. Respiratory adaptations in diving mammals. *Respir Physiol* **54**, 269-294, doi:10.1016/0034-5687(83)90072-5 (1983).

90 Patrickios, C. S. & Yamasaki, E. N. Polypeptide amino acid composition and isoelectric point. II. Comparison between experiment and theory. *Anal Biochem* **231**, 82-91, doi:10.1006/abio.1995.1506 (1995).

91 Spassov, V. Z. & Yan, L. A fast and accurate computational approach to protein ionization. *Protein Sci* **17**, 1955-1970, doi:10.1110/ps.036335.108 (2008).

92 Spassov, V. Z. & Yan, L. pH-selective mutagenesis of protein-protein interfaces: in silico design of therapeutic antibodies with prolonged half-life. *Proteins* **81**, 704-714, doi:10.1002/prot.24230 (2013).
